# Persistent Epigenetic Reprogramming Is Associated With Contrasting Drought‐Response Strategies in Citrus Scion–Rootstock Systems

**DOI:** 10.1111/ppl.71113

**Published:** 2026-09-22

**Authors:** Monique Ayala Araújo da Silva, Fredson dos Santos Menezes, Iris Sammarco, Lucas da Silva Costa, Claudia Fortes Ferreira, Walter dos Santos Soares Filho, Fabienne Micheli, Maurício Antônio Coelho‐Filho, Abelmon da Silva Gesteira

**Affiliations:** ^1^ Departamento de Biologia, Centro de Genética e Biologia Molecular Universidade Estadual de Santa Cruz Ilhéus Bahia Brazil; ^2^ Institute of Botany, Czech Academy of Sciences Průhonice Czech Republic; ^3^ Embrapa Mandioca e Fruticultura Cruz das Almas Bahia Brazil; ^4^ CIRAD—UMR AGAP Montpellier France; ^5^ UMR AGAP Institut, Univ Montpellier, CIRAD, INRAE, Institut Agro Montpellier France

**Keywords:** citrus resilience, DMRs, DNA methylation, drought stress memory, epigenetic regulation, rootstock‐scion interactions, WGBS

## Abstract

Recurrent drought limits the productivity of perennial crops such as citrus, whose tolerance to water deficit largely depends on scion–rootstock interactions. Although physiological responses to recurrent drought are well documented, the epigenetic states associated with these responses remain poorly understood, limiting the use of epigenetic variation to improve drought resilience. Here, we investigated whole‐genome DNA methylation profiles and transcriptional responses in “Pera‐D6” sweet orange (LP) grafted onto two rootstocks with contrasting physiological strategies for coping with water deficit: “Rangpur” lime (RL), associated with dehydration avoidance through enhanced water acquisition, and “Sunki Maravilha” mandarin (SM), associated with tighter regulation of transpiration and physiological adjustments under stress. Grafted plants were exposed to three cycles of water deficit followed by rehydration. In the scion, we detected rootstock‐dependent methylation dynamics, with LP/RL exhibiting broader patterns of methylation change and LP/SM showing a more targeted response. In the LP/SM combination, drought‐associated differentially methylated regions were linked to genes involved in photosynthesis, stomatal regulation, and transcriptional processes. For several stress‐responsive genes, promoter methylation changes coincided with qualitative trends in gene expression; however, no significant methylation–expression correlations were detected after multiple‐testing correction. These findings indicate that recurrent drought is associated with rootstock‐dependent persistent DNA methylation patterns with potential functional relevance. Overall, the results highlight the importance of considering epigenetic variation in studies of drought response and suggest that rootstock selection may play a role in shaping regulatory responses to water deficit in citrus.

## 1 Introduction

The increasing frequency and severity of drought events pose major challenges to plants' adaptation and maintenance of agricultural productivity. The global area affected by drought has expanded significantly, especially in the last 5 years (Gebrechorkos et al. 2025). Projections indicate that by 2100 the extent of drought‐affected regions may increase by up to 28.6%, potentially leading to crop yield reductions of as much as 90% (Li et al. 2009; Spinoni et al. 2020).

Rising temperatures and decreasing precipitation intensify the frequency of drought events and directly affect crop productivity, impacting food security and the global economy (Naumann et al. 2018; Spinoni et al. 2020). To reach their full development and reproductive potential, plants depend on ideal environmental conditions. However, environmental fluctuations can cause metabolic imbalances that limit their growth, although metabolic plasticity allows some adaptation to seasonal variations (Shao et al. 2008).

Throughout evolution, plants have developed the ability to perceive and respond to environmental stresses, activating tolerance and adaptation mechanisms even in response to the earliest signals of stress (Pandey et al. 2017). Prior exposure to water deficit events can induce adaptive responses that prepare the plant for future drought episodes, a phenomenon known as stress priming (Imadi et al. 2016; Neves et al. 2017; Hilker and Schmülling 2019a, 2019b; Li et al. 2019; Sousa et al. 2022). During post‐stress recovery, plants retain information from prior stress exposure, enabling faster and more robust responses to subsequent drought events. This phenomenon, referred to as “stress memory,” has been described as a sophisticated adaptive mechanism based on epigenetic modifications in several plant species, though its molecular basis in perennial grafted crops remains poorly characterized (Hilker et al. 2016; Neves et al. 2017; Virlouvet et al. 2018; Friedrich et al. 2018; Hilker and Schmülling 2019a, 2019b; Sousa et al. 2022). It is important to distinguish between these two related but distinct phenomena: stress priming refers to the enhanced responsiveness observed during subsequent stress exposure, whereas stress memory refers to the molecular and epigenetic states retained after stress relief and recovery. In the context of the present study, drought stress memory refers specifically to persistent DNA methylation states detected in the scion 1 week after the final drought–recovery cycle following repeated water deficit exposure. This operational definition reflects the post‐recovery sampling strategy adopted here and does not imply that the observed methylation patterns are causally responsible for the physiological acclimation observed during successive drought cycles.

Among the epigenetic mechanisms involved in stress memory, DNA methylation has been associated with changes in gene expression in plants under water deficit conditions. Studies show that water deficit can induce either an increase or a decrease in DNA methylation, influencing plant adaptation to adverse conditions (Liang et al. 2014; Li et al. 2016; Sallam et al. 2020; Akhter et al. 2021; Naderi et al. 2020; Zi et al. 2024). In rice, drought memory is established after mild cycles of drought and rehydration, mediated by phytohormones, long non‐coding RNAs (lncRNAs), and DNA methylation (Li et al. 2019). In maize, DNA methylation regulates the expression of drought‐responsive genes and affects alternative splicing. Furthermore, these methylation patterns can be transmitted across generations. In *Medicago ruthenica* , drought reduced global methylation levels by 4.41% (Zi et al. 2024), while in *Populus trichocarpa* under water deficit, 10.04% of all cytosines were methylated compared to 7.75% under irrigated conditions (Liang et al. 2014). Additionally, in wheat cultivars with contrasting drought tolerance, such as the more tolerant “Bolani” and the more sensitive “Sistan” varieties, it was observed that more tolerant plants exhibit a favorable balance toward methylation compared to more drought‐sensitive plants (Naderi et al. 2020).

Epigenetic modifications such as DNA methylation, post‐translational histone modifications, and non‐coding RNAs (ncRNAs) modulate gene expression in response to adverse conditions. DNA methylation is an epigenetic mark often associated with transcriptional repression, playing a crucial role in controlling plant genomic activity. These modifications may occur locally, affecting specific loci, or globally, altering the entire genome stability, chromatin structure, and gene expression patterns (Duan et al. 2018). DNA methylation patterns are dynamic and sensitive to environmental changes, varying according to tissue, developmental stage, and genotype, providing an adaptive advantage in a scenario of increasing climate instability (Zhu 2009; Schulz et al. 2014; Kim and Zilberman 2014; Yong‐Villalobos et al. 2015; Garg et al. 2015; Neves et al. 2017; Li et al. 2018; Colicchio et al. 2018; Sousa et al. 2022). Studies have shown that drought‐induced alterations in the methylome are associated with differences in plant responses to water deficit, with more tolerant scion/rootstock combinations exhibiting a more stable methylome, which is associated with improved recovery and adaptation under stress conditions (Neves et al. 2017; Santos et al. 2020; Sousa et al. 2022).

Citrus crops, cultivated in tropical, subtropical, and temperate regions, stand out as one of the main fruit crops, with significant global economic impact (FAOSTAT 2019; Khonglah et al. 2019). Drought is one of the primary abiotic factors threatening citrus cultivation, affecting tree growth, development, and fruit quality, resulting in a decline in orchard productivity (Osakabe et al. 2014; Nakashima et al. 2014; Xiong et al. 2024; Dohale et al. 2024). An important strategy to mitigate the negative effects of drought in citrus production and improve water use efficiency is the use of rootstocks (Mudge et al. 2009; Garcia‐Sanchez et al. 2007). Rootstocks induce physiological changes that affect leaf water potential, stomatal conductance, and hydraulic conductance (Garcia‐Sanchez et al. 2007; Rodríguez‐Gamir et al. 2011). Depending on the intrinsic characteristics of the rootstock, plants may adopt different drought survival strategies, such as dehydration avoidance or tolerance (Santana‐Vieira et al. 2016; Neves et al. 2017).

According to Santana‐Vieira et al. (2016), the citrus rootstocks *Citrus limonia* Osbeck (“Rangpur” lime) and *C. sunki* (Hayata) hort. ex Tanaka (“Sunki Maravilha”) exhibit contrasting responses to water deficit. “Rangpur” lime predominantly adopts a dehydration avoidance strategy, sustaining vegetative growth even under unfavorable conditions, whereas “Sunki Maravilha” exhibits a dehydration tolerance strategy, mitigating water loss through reduced transpiration and allocating resources to survival mechanisms at the expense of growth during drought stress. Building on these observations, Neves et al. (2017, 2018) further investigated the physiological responses of “Valencia” sweet orange grafted onto these contrasting rootstocks, subjecting plants to up to three successive cycles of water deficit followed by rehydration. Their results showed that recurrent drought exposure induces adaptive changes in photosynthetic performance, consistent with the occurrence of physiological memory associated with water deficit. Moreover, these responses varied according to the scion/rootstock combination, with different magnitudes of responses observed during subsequent drought events.

Building on these findings, Sousa et al. (2022) investigated whether this acquired physiological memory could be stored and transmitted through buds, that is, whether the history of drought exposure could generate heritable epigenetic effects via grafting. To address this question, buds from “Valencia” sweet orange plants previously exposed to up to three drought cycles on different rootstocks were collected and grafted onto “Swingle” citrumelo. The results showed that both the number of prior drought cycles and the origin of the bud (i.e., the scion/rootstock combination) significantly affected the physiological performance of plants regenerated from previously acclimated buds, suggesting that stress‐induced epigenetic states may be maintained and transmitted through grafting. Notably, buds derived from “Valencia” grafted onto “Sunki Maravilha” retained a dehydration tolerance strategy, whereas those derived from “Valencia” grafted onto “Rangpur” lime preserved a drought avoidance profile.

These findings represent an important step toward understanding drought‐associated acclimation processes in citrus and highlight the potential role of epigenetic mechanisms, such as DNA methylation, in long‐term stress adaptation. However, a key gap remains: whether contrasting scion/rootstock combinations establish distinct and reproducible methylation configurations after recurrent drought exposure, and whether such configurations are associated with the differential physiological behavior previously described for these genotypes. To date, no study has characterized genome‐wide methylation profiles at base resolution in grafted citrus subjected to repeated drought–recovery cycles, nor investigated how rootstock identity shapes persistent epigenetic states in the scion. While previous studies in citrus relied on low‐resolution MSAP to detect methylation changes under recurrent drought (Neves et al. 2017; Sousa et al. 2022), this approach does not allow the identification of specific genomic contexts, regulatory regions, or candidate genes associated with the epigenetic response to water deficit.

Rather than capturing dynamic methylation changes during acute stress imposition, this study was specifically designed to characterize persistent epigenetic states established after recurrent drought–recovery cycles, a complementary and ecologically relevant approach that reflects the cumulative epigenetic landscape experienced by plants under field‐like conditions of recurring water deficit. Based on this framework, we hypothesize that contrasting scion/rootstock combinations exposed to recurrent drought cycles exhibit distinct persistent DNA methylation configurations, reflected in genome‐wide profiles and gene‐associated methylation patterns. To address this question, we investigated genome‐wide DNA methylation profiles using whole‐genome bisulfite sequencing (WGBS) in “Pera” sweet orange grafted onto two rootstocks with contrasting drought behavior, “Rangpur” lime and “Sunki Maravilha” mandarin, subjected to three cycles of water deficit and recovery, aiming to identify rootstock‐dependent persistent epigenetic states potentially associated with recurrent drought exposure and potentially related to previously reported physiological acclimation responses.

## 2 Materials and Methods

### 2.1 Plant Material and Experimental Design

The sweet orange “Pera D‐6 CNPMF” (LP) was used as the scion, grafted onto two rootstocks with contrasting responses to water deficit: “Rangpur” lime Santa Cruz (*Citrus limonia* Osbeck; LP/RL) and “Sunki Maravilha” mandarin ( *C. sunki* (Hayata) hort. ex Tanaka; LP/SM; Neves et al. 2017; Santana‐Vieira et al. 2016). The physiological responses of citrus scion/rootstock combinations involving these rootstocks to recurrent water deficit, including stomatal conductance, photosynthetic performance, and leaf water potential, have been extensively characterized under comparable experimental conditions in previous studies from our group (Neves et al. 2017, 2018; Santana‐Vieira et al. 2016; Sousa et al. 2022), providing the physiological framework within which the epigenetic responses investigated here are interpreted.

The experimental design consisted of two scion/rootstock combinations (LP/RL and LP/SM) under two water regimes (control and recurrent drought), with three independent biological replicates per treatment subjected to WGBS (12 libraries in total).

Plants used as budwood mother plants were grown in plastic bags containing a coconut fiber‐based substrate (Golden Mix Substrate—Amafibra) and were 8 months old after grafting at the beginning of the experiment. All plants were selected based on uniform size and vigor, exhibiting similar height and stem diameter; individuals that did not meet these criteria were discarded. Prior to the start of the experiment, plants were maintained under full irrigation (Ψm = −15 kPa) to ensure stable physiological conditions before the application of water‐deficit treatments.

Soil matric potential (Ψm) was continuously monitored and recorded in a table using IRRIGÁS BÁSICO sensors (Embrapa Technology). A threshold of −25 kPa was defined as moderate stress, used to follow the gradual depletion of soil water, while −40 kPa was defined as severe stress. Rehydration was applied when plants reached the severe water deficit threshold of −40 kPa across all drought cycles and scion/rootstock combinations. The −25 kPa threshold was used only to monitor the progression of soil water depletion, not as a rehydration trigger, ensuring that drought imposition and recovery occurred at well‐defined physiological states for each scion/rootstock combination. A summary of drought cycle durations, rehydration thresholds, recovery periods, and sampling point for each combination is provided in Table S2. The plants were then subjected to the following treatments: (i) full irrigation, considered as control (Ψm = −15 kPa); and (ii) three cycles of water deficit, with soil matric potential continuously monitored at −25 kPa (moderate stress) and −40 kPa (severe stress), with rehydration applied at the severe threshold.

**FIGURE 1:**
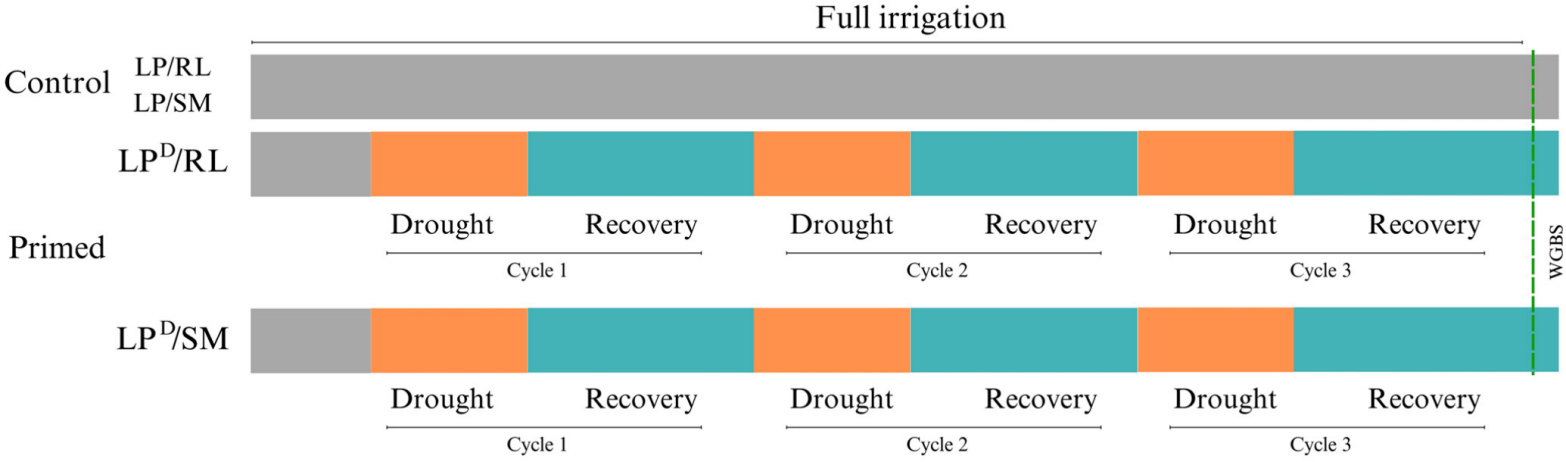
Experimental design of treatments applied to “Pêra” sweet orange plants grafted onto “Rangpur” lime (LP/RL) and “Sunki Maravilha” lime (LP/SM) rootstocks, grown under full irrigation (control) or subjected to severe water deficit followed by rehydration. Treatments were designated as LP^C^/SM and LP^C^/RL (control) and LP^D^/SM and LP^D^/RL (water deficit). Initially, all plants were maintained under full irrigation. Subsequently, they were subjected to three cycles of water deficit interspersed with rehydration periods. For LP^D^/RL, the first cycle lasted 17 days of water deficit followed by 7 days of rehydration. Soil moisture was continuously monitored using sensors to record the soil matric potential (Ψm) throughout the experiment. At the end of the experimental period, leaves were collected for DNA methylation analysis. The green dashed line in the figure indicates the timing of leaf sample collection for DNA methylation analysis.

Successive cycles of water deficit followed by recovery were imposed to induce stress memory in the citrus plants, allowing the evaluation of potential epigenetic mechanisms associated with the enhanced tolerance response to water deficit (Neves et al. 2018; Sousa et al. 2022). The treatments were designated as follows: LP^C^/RL and LP^C^/SM for control plants, and LP^D^/RL and LP^D^/SM for plants subjected to recurrent drought cycles. Each treatment consisted of six biological replicates, from which three were used for bisulfite sequencing, totaling 12 libraries. Each biological replicate consisted of leaf tissues collected from a single individual plant, representing independent biological samples. Water deficit was induced gradually as the available water in the coconut fiber‐based substrate was depleted by the plants (Figure 1). At the end of each stress cycle, all plants were rehydrated and maintained at field capacity until the start of the next water deficit cycle. To assess potential systemic effects due to the rootstock used, leaves were collected from three plants from the control group (full irrigation) and three from the group subjected to severe water deficit cycles, for each scion/rootstock combination and treatment. After a one‐week recovery period following the last water deficit cycle, leaf tissue was collected from the third and fourth fully expanded leaves counted from the shoot apex. This sampling point was chosen to capture persistent epigenetic states established after the complete drought‐recovery cycle. Only leaves showing uniform expansion, coloration, and absence of visible stress‐induced damage or senescence were included. The experiment was conducted at Viveiro Tamafe, Serra do Ramalho (13°17′34.2″ S, 43°33′41.8″ W), Bahia, Brazil, in an insect‐proof screenhouse with a transparent plastic roof, under natural light, with an estimated mean temperature of approximately 28°C and relative humidity of approximately 50%–60%.

### 2.2 Library Construction and WGBS Sequencing

#### 2.2.1 Sampling and Genomic DNA Extraction

Leaf tissues were collected as described above and immediately wrapped in aluminum foil and frozen in liquid nitrogen, then stored on dry ice for transport and subsequently kept at −80°C until DNA extraction. Three biological replicates were used per treatment. Genomic DNA extraction from leaves was carried out according to the Doyle and Doyle (1987) protocol. DNA concentration and integrity were assessed by electrophoresis on 1% agarose gel, while DNA purity (OD260/280) was determined using a Nanodrop spectrophotometer. Only samples with DNA ≥ 1 μg and concentration ≥ 10 ng μL^−1^ were considered suitable for subsequent analyses.

#### 2.2.2 DNA Library Preparation

For whole‐genome bisulfite sequencing (WGBS) library construction, 1 μg of genomic DNA was sonicated to an average fragment size of approximately 200–400 bp and subsequently used for library preparation with the Acegen Bisulfite‐Seq Library Prep Kit (Acegen), following the manufacturer's instructions. The fragmented DNA underwent end repair, 5′ phosphorylation, 3′‐dA tailing, and subsequent ligation to methylated adapters. The methylated adapter‐ligated DNA molecules were purified using Agencourt AMPure XP magnetic beads (0.8×) and subjected to bisulfite conversion using the ZYMO EZ DNA Methylation‐Gold Kit (Zymo). After conversion, DNA was amplified by PCR in a reaction containing 25 μL of KAPA HiFi HotStart Uracil+ ReadyMix (2×) and 8 bp indexed primers, each at a final concentration of 1 μM. The resulting WGBS libraries were assessed using the Agilent 2100 Bioanalyzer, quantified with the Qubit fluorometer using the Quant‐iT dsDNA HS Assay Kit (Invitrogen), with effective library concentration (> 2 nM) confirmed by qPCR, and finally sequenced on an Illumina HiSeq X Ten platform.

### 2.3 WGBS Data Analysis

#### 2.3.1 Alignment and Mapping of WGBS Reads to the *Citrus sinensis* Reference Genome

The FastQC tool (https://www.bioinformatics.babraham.ac.uk/projects/fastqc/) was used to perform basic quality statistics on raw reads. Subsequently, sequencing adapters and low‐quality sequences were removed using TrimGalore (version 0.6.7) (https://www.bioinformatics.babraham.ac.uk/projects/trim_galore/). After obtaining high‐quality reads, bisulfite‐treated sequences were mapped to the reference genome of *Citrus sinensis* , available in the NCBI Genome Assembly database (DVS_A1.0), using the Bismark software (version 0.24.0) in end‐to‐end mode with HISAT2 (version 2.2.1; Krueger and Andrews 2011). Redundant reads resulting from PCR amplification and repetitive regions in paired‐end sequences, were removed. Alignment statistics were generated, and only uniquely mapped reads were retained for subsequent analyses.

#### 2.3.2 Methylation Level Calculation

Average methylation levels were calculated for each treatment and sequence context (CG, CHG, and CHH) using custom R scripts (https://github.com/iris‐sammarco/methylation‐summary‐utils). For each sample, a weighted mean methylation proportion was computed across all cytosines within a given context as follows:

$$\text{Weighted mean}=\frac{\sum i\left(m_{i}\times c_{i}\right)}{\sum i\;c_{i}}$$

where $m_{i}$ is the methylation proportion at cytosine *i* (ratio of methylated reads to total reads) and $c_{i}$ is the total coverage at that site (sum of methylated and unmethylated reads). This weighting accounts for variation in sequencing depth among sites and yields a robust estimate of genome‐wide methylation (Schulz et al. 2014). To assess the reproducibility of WGBS data and evaluate inter‐sample variation, principal component analysis (PCA) was performed on CpG sites with a sequencing depth of ≥ 10× across all samples, using an orthogonal transformation to convert methylation levels into linearly uncorrelated principal components. Weighted mean methylation levels were summarized per biological replicate and used to test for differences among treatments (LP^C^/RL, LP^D^/RL, LP^C^/SM and LP^D^/SM) within each sequence context. The normality of residuals was assessed using the Shapiro–Wilk test. As all data met the assumption of normality (*p* > 0.05), one‐way ANOVA was performed to evaluate the effect of treatment within each sequence context. All analyses and visualizations were performed in R software.

#### 2.3.3 Differentially Methylated Regions (DMRs) Analysis

The software metilene (version 0.2–8) was used to identify differentially methylated regions (DMRs) in pairwise comparisons between treatments within scion/rootstock combinations, and between scion/rootstock combinations within treatments, using as input files cytosines filtered for coverage ≥ 5. Prior to DMR analysis, cytosines were separated by sequence context (CG, CHG, and CHH) using custom scripts, and metilene was run independently for each context, adapting the software to the three‐context plant methylation landscape. The same parameters were applied across all sequence contexts (CG, CHG, CHH), following approaches reported in comparable plant WGBS studies.

The default parameters described by Jühling et al. (2016) were used, including a mean methylation difference greater than 0.1, a maximum distance of 300 bp between consecutive cytosines within a DMR, and a minimum of five cytosines per DMR. Only the DMRs with a *q*‐value cutoff < 0.05, based on the default *p* value of the Mann–Whitney *U* test, were considered significant.

#### 2.3.4 Genomic Distribution, Enrichment, and Density of DMRs

To characterize the genomic distribution of DMRs, we quantified their overlap with annotated genomic features, including promoters, gene bodies, exons, and transposable elements (TEs; https://github.com/iris‐sammarco/DMR‐annotation‐and‐enrichment). Genomic coordinates for these features were obtained from the *Citrus sinensis* cv. Valencia DVS_A v1.0 annotation (Wu et al. 2022). Promoter regions were defined as 2 kb upstream of the transcription start site (TSS) and their length was adjusted, when necessary, to avoid overlap with adjacent genes.

TE annotations were generated in this study from the same genome using the EDTA pipeline (version 2.1.0; Ou et al. 2019). Overlaps were calculated for all pairwise comparisons using BEDTools (version 2.31.1) with the intersect function (Quinlan and Hall 2010). DMR counts overlapping each feature were then summarized and visualized using ggplot2 (version 4.0.0; Wickham 2016) in R, both (i) across all comparisons and (ii) separately for the four main biological contrasts: comparisons between control and water deficit treatments within each scion/rootstock combination, and comparisons between different scion/rootstock combinations within the same treatment.

Because exons largely overlap with gene bodies, they were excluded from subsequent DMR enrichment and density analyses to avoid statistical redundancy and inflated overlap estimates (https://github.com/iris‐sammarco/DMR‐annotation‐and‐enrichment). Thus, analyses focused on promoters, gene bodies, and TEs. Feature coordinates were merged using the reduce() function from the GenomicRanges R package to prevent redundant overlaps (version 1.58.0; Lawrence et al. 2013). For each DMR dataset (CG, CHG, and CHH contexts), we performed enrichment analyses focused on the four main comparisons.

The number of base pairs overlapping both DMRs and each genomic feature were compared to the rest of the genome using Fisher's exact tests. For each feature category (promoters, gene bodies, and TEs), a 2 × 2 contingency table was constructed with: (a) overlapping DMR base pairs, (b) DMR base pairs not overlapping the feature, (c) feature base pairs not overlapping DMRs, and (d) remaining genomic base pairs. For TEs, this analysis was first performed for all TEs combined and then independently for each TE superfamily. This approach allowed for the quantification of the total methylated sequence overlapping each feature or TE superfamily, providing a base pair‐level measure of DMR enrichment, rather than simply counting DMRs. Odds ratios (OR) from all Fisher's tests were log_2_‐transformed for visualization. *p* values were adjusted for multiple testing using the Benjamini–Hochberg false discovery rate (FDR). For both genic and TE features (including TE superfamilies), categories with FDR‐adjusted *p* values < 0.05 were classified as enriched (log_2_(OR) > 0, OR > 1) or depleted (log_2_(OR) < 0, OR < 1), respectively. Heatmaps of log_2_(OR) from Fisher's tests were generated using the *ggplot2* package in R software.

Finally, methylation levels of DMRs overlapping genes and TEs were visualized using computeMatrix and plotProfile from DeepTools version 3.5.4 (Ramírez et al. 2016) and represented as genomic density tracks (circos plots) using the R package circlize version 0.4.16 (Gu et al. 2014; https://github.com/iris‐sammarco/methylation‐and‐expression‐visualization).

#### 2.3.5 Gene Ontology (GO) and KEGG Pathway Enrichment Analysis for DMR‐Related Genes

Functional annotation of *Citrus sinensis* cv. Valencia DVS_A v1.0 proteins (Wu et al. 2022) was performed using eggNOG‐mapper v2 (Cantalapiedra et al. 2021) with default parameters. The resulting annotation file (out.emapper.annotations) was processed in TBtools v1.098 (Chen, Song, et al. 2020; Chen, Chen, et al. 2020) to extract GO and KEGG terms associated with genes overlapping DMRs. Enrichment analyses were conducted in TBtools using the “GO Enrichment” and “KEGG Enrichment Analysis” modules. The background datasets included the go‐basic.obo ontology and Plants.20211128.TBtoolsKeggBackEnd KEGG reference files, respectively. For each comparison (scion/rootstock combinations, treatment, and methylation context), the protein IDs of DMR‐associated genes were used as input. Significance of enrichment was assessed using the Benjamini–Hochberg false discovery rate (FDR < 0.05). The 10 most significantly enriched GO biological processes and KEGG pathways were selected and visualized with the “Enrichment Bar Plot” tool in TBtools. These analyses were intended to provide functional annotation of DMR‐associated genes and do not demonstrate functional activation of the enriched pathways.

### 2.4 Selection of Candidate Genes for Expression Analysis

Candidate genes for expression analysis were selected through a two‐step approach: (i) identification of DMRs in promoter or gene body regions from the WGBS data generated in this study; and (ii) genes known to be associated with key processes such as abiotic stress signaling, hormonal pathways, methylome maintenance, transcription factors linked to tolerance, and regulators of redox balance. This strategy allowed us to directly integrate methylation profiles with molecular responses potentially involved in stress memory in citrus.

### 2.5 RNA Extraction and RT‐qPCR

Total RNA was extracted using the Plant RNeasy Kit (Qiagen), following the manufacturer's recommended protocol. Starting with 3 μg of total RNA, DNase treatment was performed using the Ambion TURBO DNA‐free Kit. Subsequently, the first‐strand cDNA synthesis was conducted using the High Capacity RNA‐to‐cDNA Kit according to the manufacturer's instructions. Quantitative real‐time PCR (RT‐qPCR) was performed on the Roche LightCycler 480 system using the RL480 SYBR Green I Master Kit (Roche Applied Sciences), following the recommended protocol. For each gene evaluated, four technical replicates were performed from three biological replicates per treatment. RNA quantity and quality were assessed using the NanoDrop 2000 (Thermo Fisher Scientific).

Primers were designed using the Primer3Plus tool (http://www.bioinformatics.nl/cgi‐bin/primer3plus/primer3plus.cgi), targeting amplicons between 200 and 400 bp. The primers used for RT‐qPCR are listed in Table 1, and their reaction efficiencies ranged from 1.85 to 2.15. Reference gene expression levels of F‐BOX FAMILY PROTEIN (*FBOX*) and UBIQUITIN‐PROTEIN LIGASE 7 (*UPL7*) were used for normalization (Mafra et al. 2012). The Ct values for the reference genes remained stable, varying between 27.5 and 29 across biological and technical replicates. Relative expression levels were calculated using the ΔΔCt method (Giulietti et al. 2001), where: ΔCt = Ct (target gene) − Ct (reference gene), ΔΔCt = ΔCt (treatment) − ΔCt (control), and relative expression = 2^−ΔΔCt^. Values reported represent the mean of four technical replicates from three biological replicates per treatment.

**TABLE 1:** Primer sequences for gene analysis by RT‐qPCR.

Gene	Forward primer	Reverse primer
*RPK1*	TAGTGGCCAAGGAGAGCAAA	GGGTCCCGAAAGCTTTTCAG
*PYR1*	TAACGTCATCTCCGGGCTAC	ACGATTGAATCCATGCACCG
*MET1*	CTCCCCACATGATACCCTCC	TACTGCCGGCAAATCACCTA
*CAS*	ATCAATCCTTCTCCGGTGCA	TCCACCACGAGATTTGACGA
*NFXL2*	TTGTGTCGGTGTAGGAGCTT	GGTGATGGGCATTTGTGGTT
*NF‐YC9*	GCACAACACCAACTTGCCTA	ACATCCTCATCGGCCTTCAT
*PERK4*	TCCTGCAGCACAAGTAGTCA	ATGAAAGGCCTGGTGATGGA
*PYL8*	TCGGAATAGCAATGGAGGCA	CTAGATTCCCCTGTGCCACA
*CPK33*	ATTACGGCGCAGATATGGGA	CTGGAAATACTTGGCCACGG
*ORTHRUS2*	CACTTCAATGGCGCACGATA	CGAGAGAGACGAAGCTAGGG
*NAC029*	GAGGAGGAGAGGGAGAGTGA	TCCACCACGAGATTTGACGA
*FBOX*	TTGGAAACTCTTTCGCCACT	CAGCAACAAAATACCCGTCT
*UPL7*	CAAAGAAGTGCAGCGAGAGA	TCAGGAACAGCAAAAGCAAG

### 2.6 Methylation–Expression Correlation Analysis

To evaluate the relationship between DNA methylation and gene expression, Spearman's rank correlation coefficients (*ρ*) were calculated between methylation levels and log_2_ fold change (log_2_FC) expression values for each candidate gene and genomic context using the cor.test function in R. Samples with missing methylation values were excluded on a pairwise basis, and correlations were calculated only when at least three non‐missing sample pairs were available. To account for multiple testing, nominal *p* values were adjusted using the Benjamini–Hochberg procedure to control the false discovery rate (FDR).

### 2.7 Average DNA Methylation in Candidate Genes and Integrated Visualization With Gene Expression

Average DNA methylation levels in promoters and gene bodies of candidate genes were calculated using the “regionCounts” function from the R package methylKit (version 1.32.0), considering only cytosines with a minimum coverage of 5× to ensure data reliability (https://github.com/iris‐sammarco/methylation‐summary‐utils). Gene expression log_2_ fold changes (log_2_FC) and DNA methylation levels in promoters and gene bodies were visualized using the R package pheatmap (version 1.0.13; https://github.com/iris‐sammarco/methylation‐and‐expression‐visualization). Missing values were displayed in grey. In all heatmaps, hierarchical clustering was disabled by setting cluster_rows = FALSE and cluster_cols = FALSE. This ensures that both rows (genes) and columns (samples) are displayed in a consistent, user‐defined order, facilitating visual comparison across contexts and between expression and methylation matrices.

## 3 Results

### 3.1 Dynamics of Drought and Recovery Cycles in Contrasting Scion/Rootstock Combinations

Rehydration points were determined exclusively by the soil matric potential threshold of −40 kPa, ensuring consistency in the application of drought treatments across scion/rootstock combinations. The duration required for the soil to reach severe drought conditions differed between combinations (Figure 1). LP^D^/RL withstood progressively longer drought periods across the cycles (17, 24, and 30 days), indicating a gradual change in water‐use dynamics over successive drought events. In contrast, LP^D^/SM showed greater initial sensitivity, requiring rehydration after only 14 days in the first cycle, followed by an extended duration in the second cycle (29 days) and a slight reduction in the third (27 days), reflecting a more variable response across cycles. Notably, while LP^D^/SM required earlier rehydration than LP^D^/RL in Cycles 1 and 3, the reverse pattern was observed in Cycle 2, highlighting the distinct water‐use dynamics associated with each scion/rootstock combination.

This variation in response across the cycles suggests that the scion/rootstock combinations underwent distinct adjustments to repeated stress. LP^D^/RL showed greater initial tolerance and exhibited progressive acclimation, as indicated by the gradual increase in the time until rehydration, consistent with a robust and stable tolerance strategy. In contrast, LP^D^/SM exhibited higher sensitivity in the first cycle, followed by rapid acclimation between the first and second cycles, consistent with the activation of priming‐related mechanisms that improve stress response. However, the slight reduction in drought duration in the third cycle compared to the second indicates limitations in sustaining this adaptation.

The observed differences reflect contrasting physiological behaviors, influenced by the intrinsic characteristics of the rootstocks. Additionally, it is possible that a combination of stresses including the gradual increase in temperature inside the greenhouse may have influenced these responses. This combination of drought and heat may have shaped the response of LP^D^/SM, possibly associated with both its greater sensitivity to water deficit and the activation of adaptive mechanisms related to epigenetic memory.

### 3.2 Summary and Quality Control of Bisulfite Sequencing (WGBS)

To identify the global cytosine methylation patterns in budwood mother plants with drought stress memory, we performed whole‐genome bisulfite sequencing (WGBS). The sequencing data was deposited in the NCBI Sequence Read Archive (SRA) (https://ncbi.nlm.nih.gov/subs/sra) under accession number PRJNA1282485. In this study, an average of 45 million high‐quality paired‐end reads was generated, corresponding to 61.29 Gb of data, with an average sequencing depth of 20× (Table S1). Of these reads, 48.35% were uniquely mapped to the *Citrus sinensis* reference genome (Table S1). This mapping rate is consistent with values reported in WGBS studies of plant species with complex genomes and high repetitive content, where bisulfite conversion reduces sequence complexity and limits unique alignment efficiency. Bisulfite conversion efficiency was consistently high across all samples (average 99.47%), and unique alignment rates varied within the expected range for outbred perennial material (Table S1). To assess data reproducibility, PCA of CG methylation levels revealed distinct clustering of control and drought‐treated plants in both scion/rootstock combinations (Figure S2), supporting the reliability of the dataset for downstream methylation analyses.

### 3.3 DNA Methylation Dynamics in the Citrus Genome Under Recurrent Drought Conditions

To characterize persistent methylome states following recurrent drought–recovery cycles, we examined global DNA methylation patterns across scion/rootstock combinations.

**FIGURE 2:**
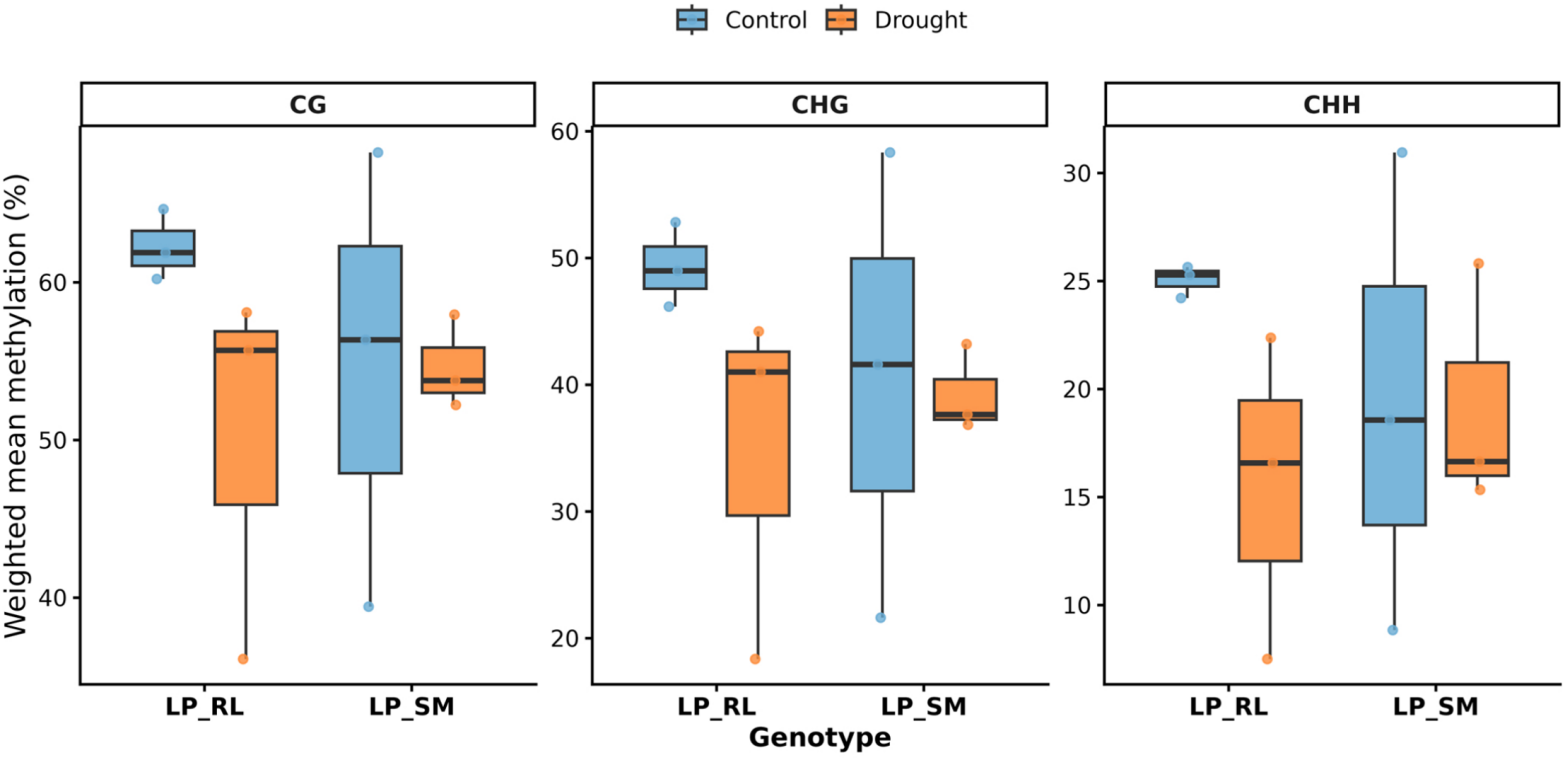
Weighted mean levels of DNA methylation in citrus scion/rootstock combinations. Boxplots show the coverage‐weighted mean percentage of methylated cytosines (mCs) in the CG, CHG, and CHH contexts in LP/RL and LP/SM under different treatments. Each dot represents one independent biological replicate, corresponding to one individual plant (*n* = 3 plants per treatment). The horizontal line within each box indicates the median, boxes represent the interquartile range, and whiskers extend to the most extreme values within 1.5 × the interquartile range. Differences among treatments were assessed separately for each methylation context using one‐way ANOVA. No statistically significant differences were detected among treatments in any methylation context (one‐way ANOVA, all *p* > 0.49).

An average of 804 and 832 million methylated cytosines (mCs) were identified in the LP/RL and LP/SM scion/rootstock combinations, respectively. Weighted average methylation levels varied among contexts, with CG methylation generally higher than CHG and CHH (Figure 2). Among control plants, weighted mean methylation levels tended to be slightly higher in LP^C^/RL than in LP^C^/SM across all contexts. Under drought, this pattern was less consistent, with LP^D^/SM showing comparable or higher methylation levels than LP^D^/RL in CHG and CHH contexts (Figure 2). No statistically significant differences in global methylation levels were detected between treatments in any context (ANOVA, all *p* > 0.49), indicating that drought‐induced methylation changes in this system are not detectable at the genome‐wide level. Locus‐specific methylation changes were therefore further investigated through DMR analysis.

### 3.4 DNA Methylation Profiles of Citrus Scion/Rootstock Combinations With Drought Stress Memory

**FIGURE 3:**
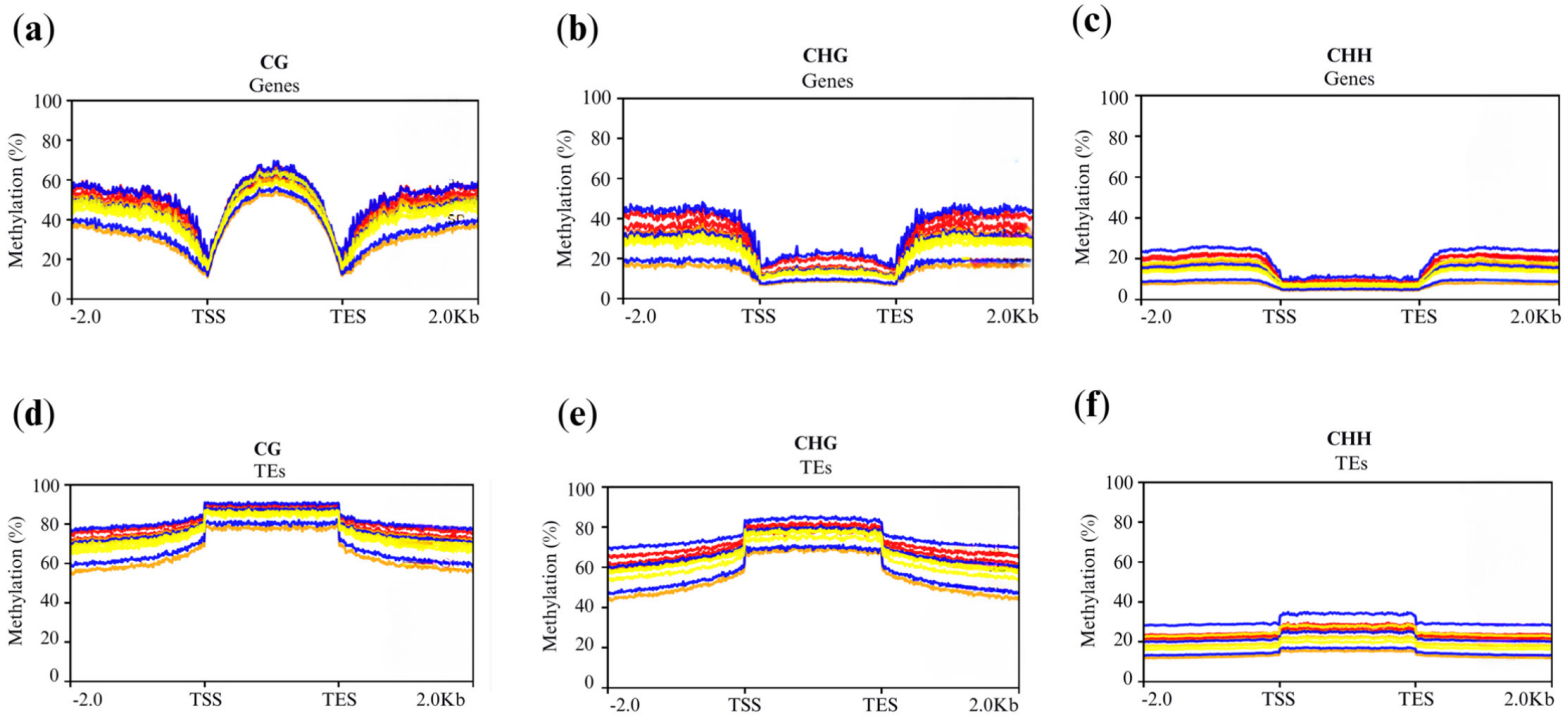
DNA methylation profiles across genes and transposable elements (TEs) in citrus scion/rootstock combinations under different treatments. Average methylation levels (%) are shown for cytosines in CG, CHG, and CHH sequence contexts across gene regions (a–c), extending from 2 kb upstream of the transcription start site (TSS), through the gene body (TSS–TES), to 2 kb downstream of the transcription end site (TES), and across TE regions (d–f) using the same layout. Curves represent individual biological replicates for four treatment/scion/rootstock combinations: LP^C^/RL (red), LP^D^/RL (orange), LP^C^/SM (blue), and LP^D^/SM (yellow), with three independent biological replicates per treatment. At each position, the plotted value represents the mean methylation level across the genes or TEs included in the analysis. No statistical tests were performed on the plotted methylation profiles.

DNA methylation levels were assessed in genes and transposable elements (TEs) across the four treatments (LP^C^/RL, LP^D^/RL, LP^C^/SM and LP^D^/SM), separately for each methylation context (Figure 3). As expected, regardless of scion/rootstock combinations or treatment, methylation levels were higher in the CG context, especially within gene bodies, compared to the 2 kb flanking regions upstream of the transcription start site (TSS) and downstream of the transcription end site (TES; Figure 3a). In contrast, CHG and CHH contexts showed lower methylation levels in both gene bodies and flanking regions (Figure 3b,c).

Overall, LP^D^/SM tended to exhibit lower DNA methylation levels in gene bodies than the other scion/rootstock combinations, whereas LP^C^/SM and LP^C^/RL generally displayed higher and broadly similar methylation levels, despite considerable overlap among biological replicates, particularly in the CHH context (Figure 3a–c). The most consistent pattern across contexts was the lower methylation levels in methylation observed in LP^D^/SM relative to the other combinations. Although variation among biological replicates was observed, particularly in the CHH context, LP^D^/SM generally exhibited lower methylation levels than the remaining scion/rootstock combinations. Similar trends were observed in both gene bodies and flanking regions across the three methylation contexts.

Analysis of methylation profiles along TEs revealed higher methylation levels in these regions than in gene bodies across all scion/rootstock combinations and methylation contexts (Figure 3d–f). In TE bodies, CG methylation levels varied among the scion/rootstock combinations, with LP^C^/SM generally exhibiting higher methylation than LP^D^/SM (Figure 3d). LP^D^/SM consistently tended to display comparatively lower methylation levels than the remaining combinations, whereas LP/RL combinations showed intermediate methylation levels with relatively small differences between control and drought‐treated plants. Similar treatment‐dependent patterns were observed in the CHG and CHH contexts, although absolute methylation levels were lower than those observed in the CG context (Figure 3e,f).

### 3.5 Functional Distribution and Enrichment of DMRs Across Contexts and Genomic Regions

**FIGURE 4:**
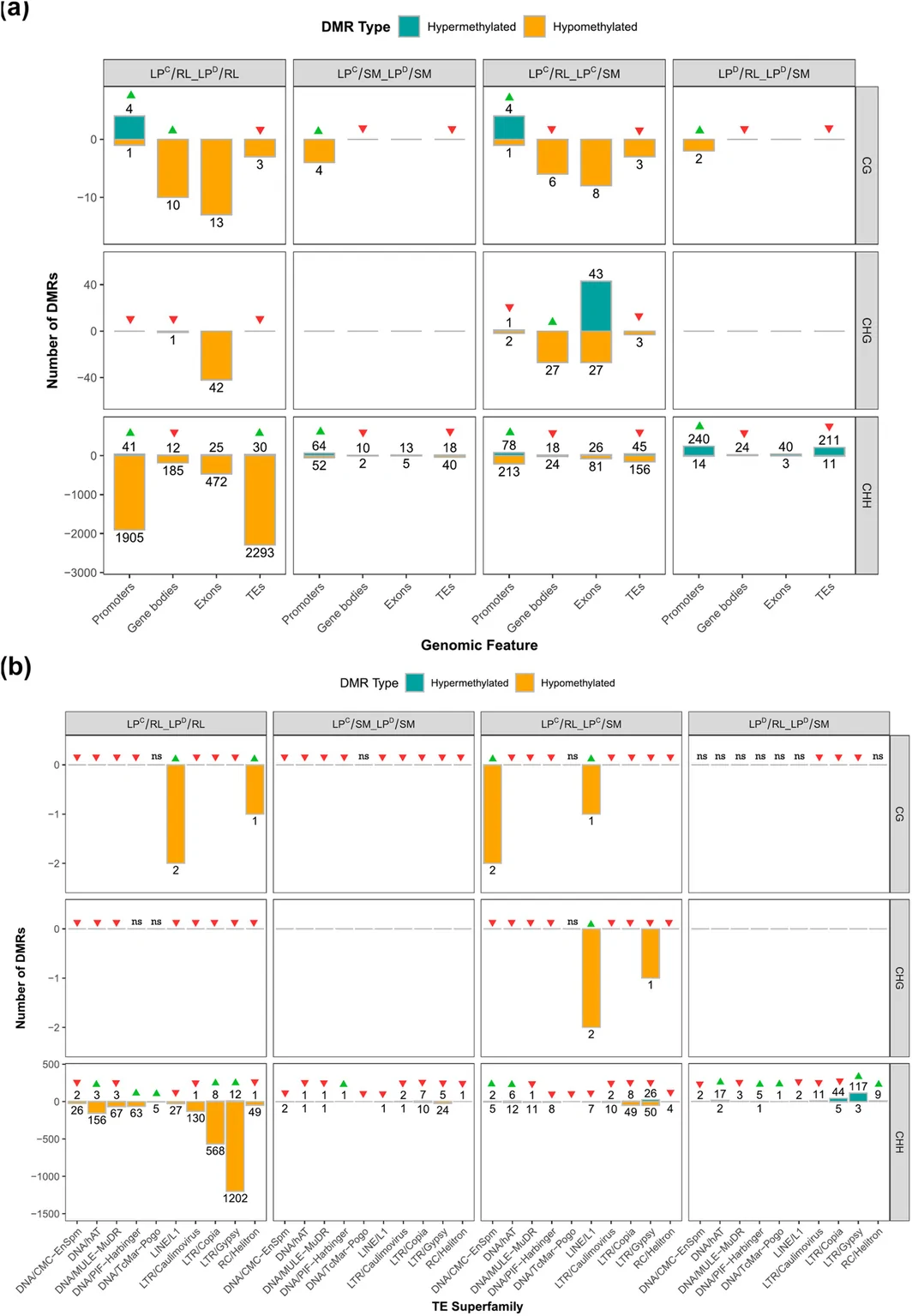
Distribution of differentially methylated regions (DMRs) across methylation contexts and genomic features in different comparisons of scion/rootstock combinations and treatments. (a) Barplots show the number of hypermethylated (blue) and hypomethylated (orange) DMRs assigned to promoters, gene bodies, exons, and transposable elements (TEs) in the three methylation contexts (CG, CHG, and CHH). Each panel represents a comparison between scion/rootstock combinations under control and water deficit conditions (LP^C^/RL_LP^D^/RL, LP^C^/SM_LP^D^/SM, LP^C^/RL_LP^C^/SM, LP^D^/RL_LP^D^/SM). (b) Barplots show the numbers of hypermethylated and hypomethylated DMRs assigned to each TE superfamily across the three methylation contexts and the same pairwise comparisons. Symbols above bars indicate enrichment status based on Fisher's exact test with correction for multiple testing: ▲ significantly overrepresented (enriched); ▼ significantly underrepresented (depleted); ns not significant (Fisher's exact test, FDR‐adjusted *p* < 0.05).

To analyze the distribution and functional relevance of DMRs in citrus plants with drought stress memory, we considered four genomic regions: promoters, gene bodies, exons, and TEs. Across all comparisons, DMRs were predominantly observed in the CHH context, particularly in hypomethylated regions, whereas CG and CHG DMRs were less frequent and more subtle. The LP/RL scion/rootstock combinations exhibited the highest number of DMRs, especially in the LP^C^/RL_LP^D^/RL (8835 DMRs) and LP^C^/RL_LP^C^/SM (1107 DMRs) comparisons, indicating extensive epigenomic remodeling. By contrast, the LP/SM scion/rootstock combinations showed fewer DMRs (407 in LP^C^/SM_LP^D^/SM) and a more balanced pattern between hypermethylation and hypomethylation, suggesting greater global methylome homeostasis or a targeted response to drought (Table 2 and Figure 4a).

**TABLE 2:** DMRs identified in each methylation context in different comparisons between scion/rootstock combinations and treatments.

Sample	CG	CHG	CHH	Total
LP^C^/RL_LP^D^/RL	12	7	8816	8835
LP^C^/SM_LP^D^/SM	5	0	402	407
LP^C^/RL_LP^C^/SM	12	17	1078	1107
LP^D^/RL_LP^D^/SM	2	0	1156	1158
LP^C^/RL_LP^D^/SM	5	5	2159	2169
LP^D^/RL_LP^C^/SM	7	4	1309	1320

DMRs were distributed preferentially in promoters and TEs, followed by gene bodies and exons, suggesting that drought‐associated and genotype‐specific methylation changes primarily target regulatory and TE regions, potentially impacting gene expression and genome stability. For instance, in LP^C^/RL_LP^D^/RL, CHH hypomethylated DMRs were most abundant in promoters (1905 DMRs) and TEs (2293), with smaller contributions from exons (472) and gene bodies (185). In the LP^C^/RL_LP^C^/SM comparison, a similar but less pronounced pattern was observed. In LP/SM, CHH DMRs showed lower numbers in all features. In CG and CHG contexts, DMRs were comparatively rare, with most changes in promoters and exons in comparisons involving LP/RL.

Fisher's exact tests confirmed non‐random localization of DMRs. In CG, DMRs were significantly enriched in promoters (log_2_OR 2.33–4.52; FDR < 0.05) and variably enriched or depleted in gene bodies and TEs. CHG DMRs were scarce but enriched in gene bodies in some comparisons, while promoters and TEs were largely depleted or untestable. In CHH, promoters were enriched (log_2_OR 0.34–0.82), gene bodies were strongly depleted (log_2_OR −3.33 to −5.30), and TEs showed mild enrichment or depletion depending on the comparison. Analysis of DMR density mirrored these patterns, with promoters harboring the highest DMR density, followed by TEs and gene bodies (Table S4). Collectively, these results indicate that methylation changes in citrus under drought are context‐ and feature‐dependent, preferentially targeting regulatory regions, with CHH methylation mediating the most dynamic changes and CG/CHG methylation concentrated in genic or regulatory regions.

The analysis of DMRs associated with TEs revealed that methylation changes occurred predominantly in the CHH context, particularly in retrotransposons of the LTR/Gypsy and LTR/Copia families (Figure 4b and Table S5). Other families, including DNA transposons such as DNA/hAT, DNA/PIF, and DNA/TcMar, also exhibited DMRs, although to a lesser extent. Notably, the association between DMRs and specific TE superfamilies varied depending on the methylation context and comparison. In the CHH context, many TE superfamilies were significantly depleted in DMRs across most comparisons. The low number of DMRs detected in LP^C^/SM_LP^D^/SM compared to LP^C^/RL_LP^D^/RL is potentially more functionally relevant and locus‐specific epigenetic response in the LP/SM combination, where methylation changes are concentrated in fewer but potentially more functionally relevant genomic regions. For this comparison, enrichment analyses of TE superfamilies should be interpreted as exploratory given the limited number of DMRs available for statistical testing. This pattern of depletion was particularly evident in LP^C^/SM_LP^D^/SM and LP^C^/RL_LP^C^/SM, where almost all TE superfamilies were depleted. The only exceptions were enrichments observed for DNA/PIF Harbinger in LP^C^/SM_LP^D^/SM, and for DNA/CMC and DNA/hAT in LP^C^/RL_LP^C^/SM (Fisher's exact test, FDR‐adjusted *p* < 0.01; Figure 4b and Table S5).

By contrast, in LP^C^/RL_LP^D^/RL and LP^D^/RL_LP^D^/SM, several TE superfamilies were significantly enriched in CHH DMRs, particularly DNA transposons (DNA/PIF, DNA/TcMar, and DNA/hAT) and LTR retrotransposons (LTR/Gypsy and LTR/Copia in LP^C^/RL_LP^D^/RL). In the CG and CHG contexts, DMRs were comparatively rare and showed limited overlap with TEs. Only a few TE superfamilies, such as LINE/L1 and RC/Helitron, were enriched in CG DMRs in specific comparisons, whereas others were depleted in CG and CHG in LP^C^/RL_LP^C^/SM. These results indicate that CG and CHG methylation changes occurred preferentially in genic or regulatory regions (Figure 4b), while CHH methylation changes occasionally targeted specific TE superfamilies.

### 3.6 Genome‐Wide DNA Methylation Across Citrus Chromosomes

**FIGURE 5:**
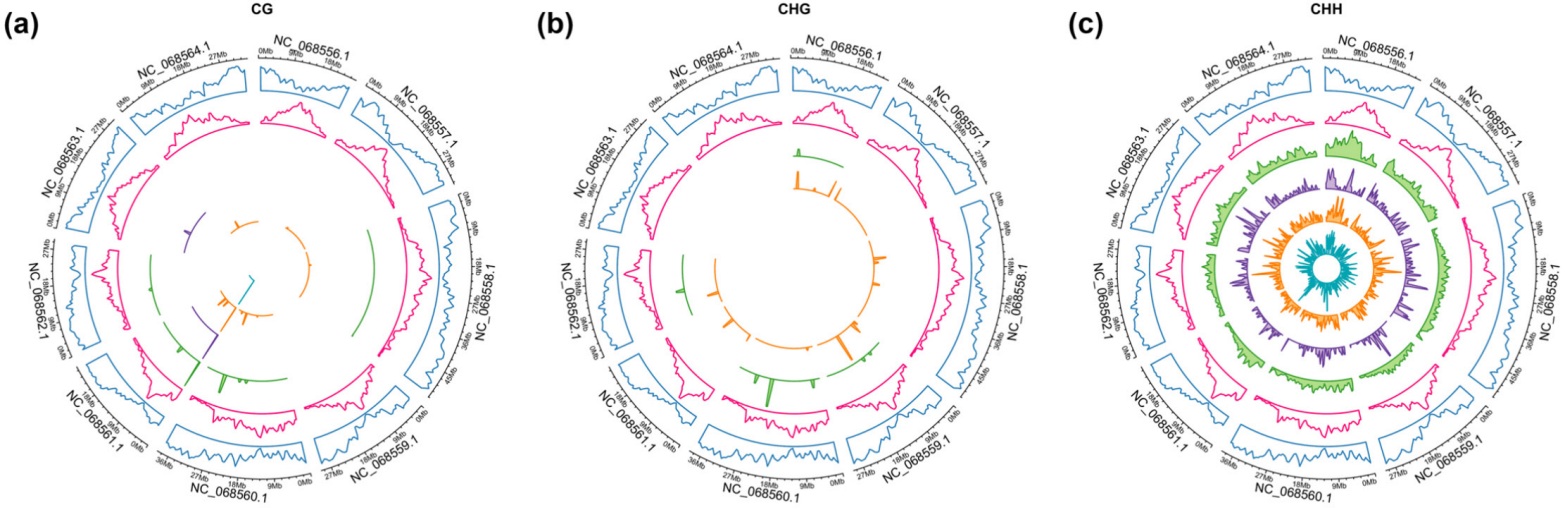
Circos plots illustrating genomic features and differentially methylated regions (DMRs) densities across citrus chromosomes. From the outermost to the innermost tracks: Gene density (blue) and transposable element (TE) density (red) are shown for each chromosome. Inner tracks represent the density of DMRs for the following comparisons: LP^C^/RL_LP^D^/RL (green), LP^C^/SM_LP^D^/SM (purple), LP^C^/RL_LP^C^/SM (orange) and LP^D^/RL_LP^D^/SM (light blue). Panels (a–c) correspond to CG, CHG, and CHH methylation contexts, respectively. No statistical tests were performed on the densities shown.

In citrus, gene density tends to be concentrated at the terminal regions of the chromosomes, whereas centromeric regions show lower gene density. On the other hand, TE density is higher in centromeric regions, reflecting the typical genomic organization with TE accumulation in heterochromatic regions, which are associated with gene transcriptional repression (Figure 5). This pattern is consistent across all nine analyzed chromosomes. The genomic distribution of DMRs across citrus chromosomes revealed distinct spatial patterns that varied according to methylation context and treatment/genotype comparisons. In the CG context, DMRs were generally concentrated in specific chromosomal regions, with a consistently pronounced enrichment in the telomeric areas of chromosome NC_068561.1 (Figure 5a). This region exhibits moderate gene and TE density and showed a consistently pronounced enrichment of CG DMRs. In contrast, CHH‐context DMRs displayed a broader and more intense distribution across all chromosomes, particularly in comparisons involving the LP/RL scion/rootstock combinations (Figure 5c). This diffuse pattern was observed predominantly in comparisons involving the LP/RL scion/rootstock combinations. Notably, a substantial overlap was observed between CHH DMRs and TE‐rich regions, consistent with the overlap between TEs and stress‐responsive epigenetic reprogramming. Comparisons involving the LP/SM scion/rootstock combinations, especially under drought conditions, showed a more localized DMR distribution, with fewer widespread changes along the chromosomes.

### 3.7 Functional Enrichment Analysis of DMRs in Different Methylation Contexts

**FIGURE 6:**
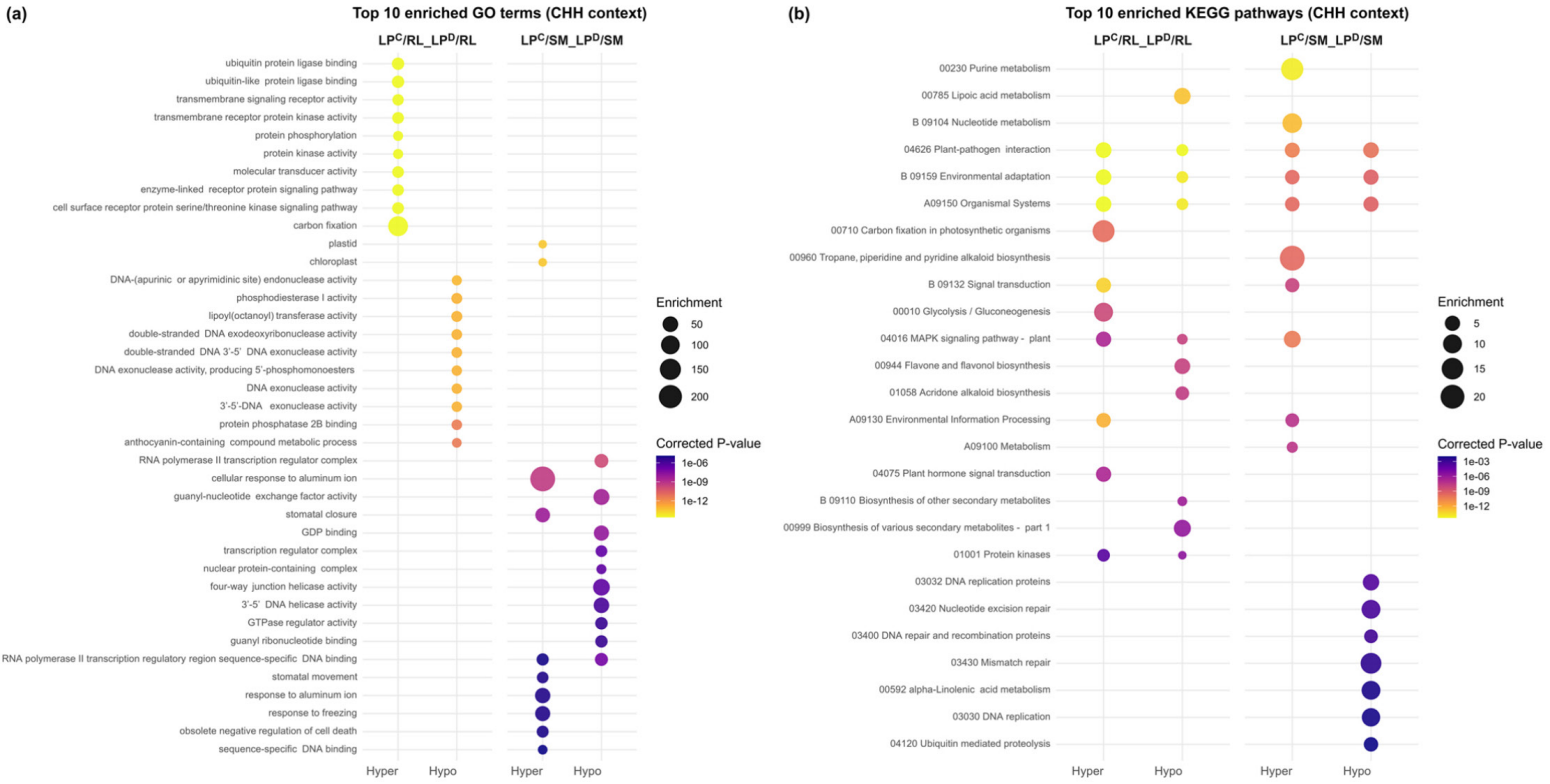
Enrichment analysis of differentially methylated regions (DMRs) in the CHH context. (a) Gene Ontology (GO) enrichment analysis of DMR‐associated genes. Dot size reflects the enrichment score (overrepresentation compared to the genomic background), and dot color shows the FDR‐corrected *p* value (yellow = highly significant, blue = less significant). Only the top 10 terms per comparison and direction (hypermethylated vs. hypomethylated) are shown. (b) KEGG pathway enrichment analysis of DMR‐associated genes. Dot size represents the enrichment factor (ratio of DMR‐associated genes in a pathway to all genes in that pathway), and dot color shows the FDR‐corrected *p* value (yellow = highly significant, blue = less significant). Only the top 10 pathways per comparison and direction are shown.

To investigate the biological processes (BP), molecular functions (MF), and cellular components (CC) potentially affected by drought‐induced epigenetic alterations in each scion/rootstock combination, Gene Ontology (GO) enrichment analysis was performed based on the DMRs identified in the LP^C^/RL_LP^D^/RL and LP^C^/SM_LP^D^/SM comparisons. The analyses were conducted separately for hypermethylated and hypomethylated DMRs in each methylation context. In the main text, we present only the results for the CHH context, which exhibited the highest number of DMRs (Figure 4a). For clarity, only the 10 most significantly enriched GO terms are shown (Figure 6a).

In the LP^C^/RL_LP^D^/RL comparison, hypermethylated DMRs were primarily enriched in biological processes associated with carbon fixation and receptor‐mediated signaling, including receptor serine/threonine kinase activity, enzyme‐linked receptor signaling, and molecular transducer activity (Figure 6a). In contrast, hypomethylated DMRs were predominantly associated with DNA repair and antioxidant‐related processes, including endo/exonuclease activities and anthocyanin metabolism, suggesting that loci involved in genome maintenance and oxidative stress responses were preferentially represented among hypomethylated regions.

In the LP^C^/SM_LP^D^/SM comparison, hypermethylated DMRs were enriched in GO terms associated with stomatal regulation, abiotic stress responses, and cell survival, including stomatal closure and negative regulation of cell death (Figure 6a). Hypomethylated DMRs were mainly associated with transcriptional regulation and chromatin remodeling, including helicase activity and RNA polymerase II regulatory complexes. Notably, none of the enriched GO terms overlapped with those identified in LP/RL, indicating distinct genotype‐dependent functional responses to recurrent drought.

### 3.8 Functional Analysis of KEGG Pathways for Hypomethylated and Hypermethylated DMRs

To elucidate the potential biological implications of the observed epigenetic modifications, enrichment analysis of metabolic and signaling pathways was performed based on KEGG terms associated with hypomethylated and hypermethylated DMRs in the CHH context. The most significantly enriched KEGG pathways (FDR < 0.05) are summarized in a dot plot (Figure 6b). Functional enrichment revealed clear contrasts between the pathways associated with hypermethylated and hypomethylated DMRs in the LP^C^/RL_LP^D^/RL scion/rootstock combinations (Figure 6b). Hypermethylated DMRs were mainly enriched in pathways associated with signal transduction, environmental adaptation, and stress‐related metabolism, including plant‐pathogen interaction and tropane, piperidine, and pyridine alkaloid biosynthesis (Figure 6b). Together, these pathways suggest that DNA hypermethylation is associated with regulatory processes involved in stress perception and response.

In contrast, hypomethylated DMRs were predominantly associated with pathways linked to genome maintenance and DNA repair mechanisms, including DNA replication proteins, nucleotide excision repair, mismatch repair, and DNA repair and recombination proteins. Additional enrichment was detected in ubiquitin‐mediated proteolysis and α‐linolenic acid metabolism, indicating that DNA hypomethylation was associated with pathways involved in genomic maintenance, protein turnover, and lipid‐derived signaling under stress conditions. Similar patterns were observed for the LP^C^/SM_LP^D^/SM scion/rootstock combinations (Figure 6b). Hypermethylated DMRs were enriched in tropane, piperidine, and pyridine alkaloid biosynthesis, plant MAPK signaling, and other categories within metabolism, signal transduction, and environmental adaptation. Conversely, hypomethylated DMRs showed significant enrichment in pathways such as mismatch repair, nucleotide excision repair, DNA replication, ubiquitin‐mediated proteolysis, and α‐linolenic acid metabolism and defense‐related processes.

**FIGURE 7:**
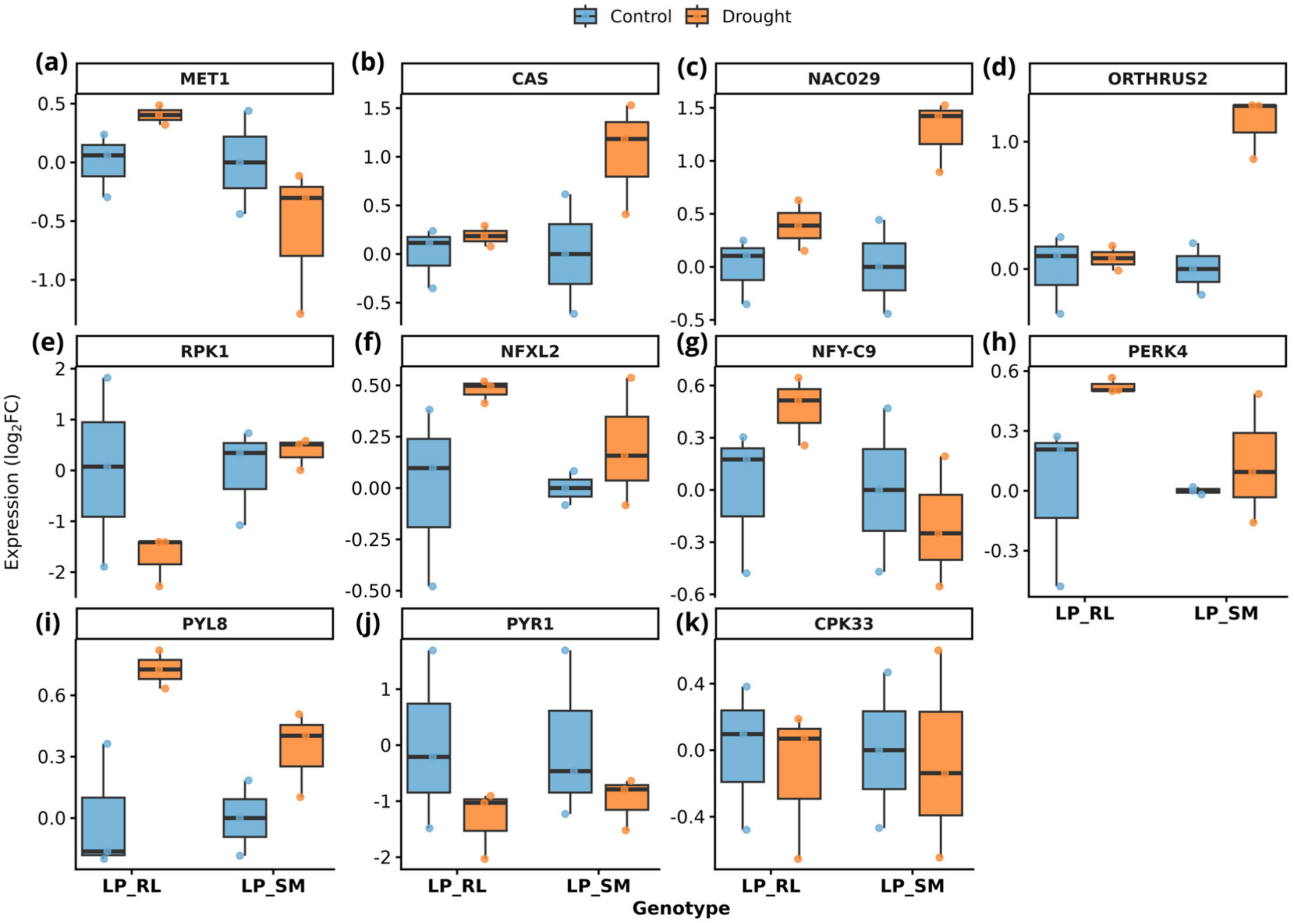
Expression of candidate genes involved in epigenetic regulation in LP/RL and LP/SM grafting combinations subjected to cycles of water deficit. Boxplots show gene‐expression values on a log_2_ fold change (log_2_FC) scale relative to the corresponding control condition. Each dot represents one biological replicate (*n* = 3 independent biological replicates per treatment). Boxes indicate the interquartile range, horizontal lines indicate the medians, and whiskers extend to the most extreme values within 1.5× the interquartile range. Pairwise comparisons were performed separately for each gene for (i) control versus drought treatments within each genotype (LP^C^/RL_LP^D^/RL, and LP^C^/SM_LP^D^/SM) and (ii) the two scion/rootstock combinations under the same treatment (LP^C^/RL_LP^C^/SM, and LP^D^/RL_LP^D^/SM). Depending on the Shapiro–Wilk normality results, comparisons were performed using Welch's *t*‐test or Wilcoxon rank‐sum test. *p* values were adjusted across genes within each comparison using the Benjamini–Hochberg method. No statistically significant differences were detected after FDR adjustments (*p*adj ≥ 0.05 for all comparisons).

**FIGURE 8:**
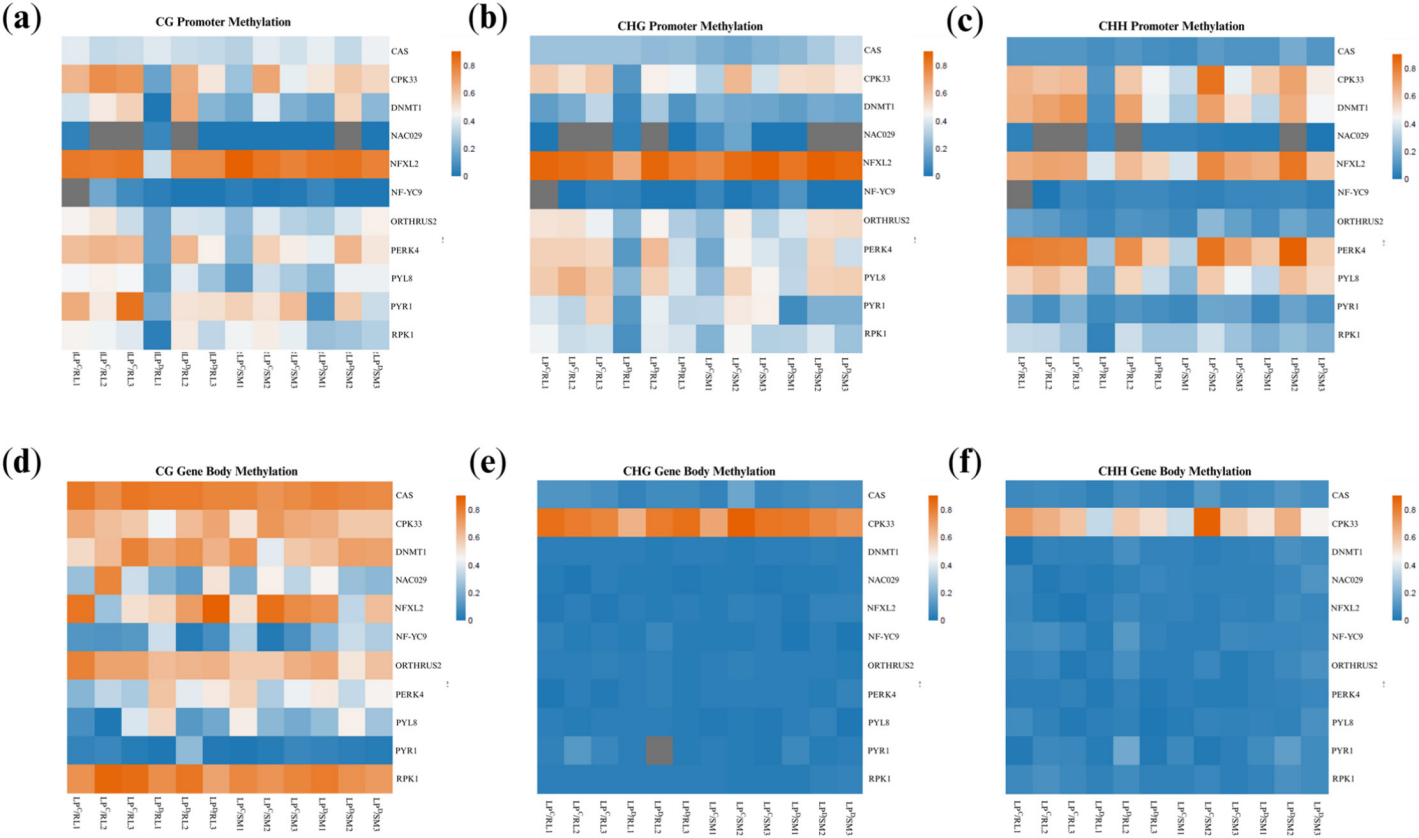
DNA methylation levels in selected genes associated with drought response in grafted citrus plants. Heatmaps show mean methylation proportions in the CG, CHG, and CHH contexts across candidate promoter regions (a–c) and candidate gene‐body regions (d–f). Rows represent candidate genes, and columns represent individual biological replicates from four grafting combinations (LP^C^/RL, LP^D^/RL, LP^C^/SM and LP^D^/SM) (*n* = 3 biological replicates per combination). Methylation was calculated as the proportion of methylated cytosines relative to total cytosine coverage across each promoter or gene‐body region. Blue indicates lower methylation proportions and orange indicates higher methylation proportions. Color scales were set separately for each heatmap. No statistical tests were performed for the heatmaps.

We investigated the DNA methylation and expression patterns of 11 candidate genes associated with drought signaling and epigenetic regulation to evaluate their potential contribution to genotype‐specific responses to water deficit (Figures 7 and 8). No statistically significant differences in gene expression were detected between treatments (FDR‐adjusted *p* < 0.05). Therefore, the following observations describe non‐significant but consistent expression patterns across genotypes. In the LP^C^/RL_LP^D^/RL comparison, CHH DMRs overlapped with the promoter regions of *PERK4*, *CAS*, and *NFXL2*, as well as gene bodies of *CAS* and *CPK33* (Table S6). However, these methylation changes were not associated with corresponding variation in gene expression, indicating no clear relationship between DMRs and transcriptional regulation in these loci. To facilitate the interpretation of candidate drought‐responsive loci, representative IGV snapshots of CHH DMRs located in promoter and gene‐body regions were generated for selected genes and are presented in Figure S1. These visualizations provide locus‐level support for the methylation differences identified in the genome‐wide DMR analysis. Spearman's rank correlation analyses between methylation levels and transcript abundance of all candidate genes detected no statistically significant associations after false discovery rate (FDR) correction (Table S7).

In LP/RL combinations, *MET1*, a key gene involved in CG methylation maintenance, showed a trend toward increased expression under drought (LP^D^/RL) relative to control conditions (LP^C^/RL). This pattern was accompanied by reduced promoter methylation, particularly in the CG context (Figures 7a and 8a–c), and increased CG methylation within the gene body (Figures 7a and 8d,e). In contrast, in LP/SM plants, *MET1* expression showed an opposite trend under drought, coinciding with increased CG methylation in the gene body.

Several drought‐responsive genes, including *CAS, NAC029, ORTHRUS2*, and *RPK1*, displayed higher expression levels in LP^D^/SM compared to LP^C^/SM (Figure 7b–e), whereas in LPD/RL similar but less pronounced expression changes were observed. Notably, these expression profiles were not consistently associated with changes in DNA methylation in promoter or gene body regions (Figure 8a,b).

Genes related to abscisic acid (ABA) signaling, such as *NFXL2*, *PERK4*, and *PYL8*, showed increased expression under drought in LP/SM (Figure 7e,h,i), whereas *NF‐YC9* and *PYR1* displayed the opposite trend (Figure 7f,j). In LP/RL, the genes exhibited only minor expression variation between treatments. Finally, *CPK33* expression remained stable across all scion/rootstock combinations, despite consistently high methylation levels in CHG and CHH contexts within the gene body (Figures 7k and 8e,f), suggesting constitutive or tightly regulated expression independent of methylation changes.

## 4 Discussion

Previous studies demonstrated that citrus rootstocks exhibit contrasting survival strategies under water deficit, with “Rangpur” lime favoring dehydration avoidance and sustained growth, whereas “Sunki Maravilha” displays dehydration tolerance associated with reduced transpiration and prioritization of stress protection over growth (Santana‐Vieira et al. 2016). Subsequent studies showed that recurrent drought exposure induces genotype‐dependent physiological acclimation consistent with stress memory, affecting photosynthetic performance across successive drought events (Neves et al. 2017, 2018). Further evidence suggested that these responses may be maintained and transmitted through vegetative propagation, with bud origin influencing the performance of newly established plants, supporting the involvement of epigenetic mechanisms in mediating these persistent responses (Sousa et al. 2022).

Despite these advances, the molecular basis underlying these responses remains poorly understood, particularly at the level of genome‐wide DNA methylation. Previous studies relied on low‐resolution approaches such as MSAP, which do not allow the identification of specific genomic regions or candidate genes associated with drought responses. Here, by applying whole‐genome bisulfite sequencing, we provide a genome‐wide characterization of DNA methylation patterns associated with recurrent drought exposure. The present study was specifically designed to characterize persistent epigenetic states following recurrent drought–recovery cycles, rather than to reassess the physiological responses of these combinations, which have been thoroughly documented under comparable experimental conditions by Santana‐Vieira et al. (2016), Neves et al. (2017, 2018), and Sousa et al. (2022). The physiological framework established by these studies—including measurements of stomatal conductance, leaf water potential, photosynthetic performance, and ABA levels—provides the biological context within which the epigenetic patterns described here are interpreted. Our results reveal rootstock‐dependent methylation dynamics, providing a genomic framework to interpret the contrasting physiological strategies previously described for these scion/rootstock combinations.

### 4.1 Scion/Rootstock Interaction Is Associated With Contrasting Physiological Responses to Water Deficit

Although both scion/rootstock combinations were subjected to the same water regime, they exhibited distinct temporal responses across drought cycles. LP^D^/RL showed a progressive increase in drought duration (17, 24, and 30 days across Cycles 1, 2, and 3, respectively), indicating a gradual shift in water‐use dynamics consistent with the dehydration avoidance strategy of the RL rootstock, including efficient water extraction, lower stomatal conductance, and osmolyte accumulation (Chaves et al. 2009; Tardieu 2012; Santana‐Vieira et al. 2016). In contrast, LP^D^/SM showed greater initial sensitivity (14 days in Cycle 1), followed by pronounced acclimation in Cycle 2 (29 days) and stabilization in Cycle 3 (27 days). This pattern suggests that a single priming event may be associated with epigenetic and physiological adjustments consistent with stress memory formation in LP/SM, with subsequent cycles serving to consolidate rather than amplify the established adaptive response (Neves et al. 2017).

The progressive increase in drought duration across cycles in LP^D^/RL and the rapid acclimation followed by stabilization in LP^D^/SM corroborate previous findings in citrus combinations involving the same rootstocks with a different scion variety (Neves et al. 2017), suggesting that rootstock identity is a primary determinant of drought response strategy, independent of scion genotype. While Neves et al. (2017) characterized epigenetic responses during stress application, the present study complements those findings by showing that distinct epigenetic states persisted after rehydration, consistent with the consolidation of stress memory in a rootstock‐dependent manner.

### 4.2 Locus‐Specific Methylation Reprogramming Is Associated With Contrasting Drought Response Strategies in LP/RL and LP/SM

Across both scion/rootstock combinations, CG methylation predominated over CHG and CHH, a canonical plant pattern, yet the relative distribution and stress‐induced dynamics of each context differed markedly between LP/RL and LP/SM, revealing combination‐specific methylation architectures associated with contrasting drought responses. To characterize these architectures, we first examined global methylation levels across all cytosine contexts. Despite the marked difference in drought history between control plants (maintained under full irrigation throughout) and drought‐exposed plants (subjected to three stress‐recovery cycles), global methylation levels did not differ significantly between treatments in either scion/rootstock combination (ANOVA, *p* > 0.49). This result suggests that drought‐associated responses in citrus may involve locus‐specific methylation changes rather than broad genome‐wide shifts, a pattern consistent with targeted epigenetic reprogramming (Kinoshita and Seki 2014; Hilker et al. 2016).

However, the two combinations differed markedly in their methylation profiles (Figure 2), with variations depending on the genotypic modifications (Neves et al. 2018; Auler et al. 2021; Tsaballa et al. 2021; Sousa et al. 2022). LP/RL exhibited higher basal CG methylation levels than LP/SM under control conditions and showed only subtle variation between control and drought‐exposed plants across all contexts, a pattern of global methylome homeostasis consistent with the dehydration avoidance strategy previously described for this rootstock (Santana‐Vieira et al. 2016; Neves et al. 2017). It is important to note that this global methylome homeostasis refers to the absence of significant changes in global methylation levels between control and drought‐exposed plants (ANOVA, *p* > 0.49), not to the absence of locus‐specific changes. Indeed, DMR analysis revealed that LP/RL harbored a substantially higher number of locus‐specific DMRs (8835) than LP/SM (407), indicating that the two combinations differ not only in the magnitude but also in the nature of their epigenetic responses: LP/RL undergoes widespread locus‐specific remodeling while maintaining overall methylome homeostasis, whereas LP/SM shows greater global amplitude between conditions but fewer discrete locus‐specific changes. Together, these observations indicate that the two scion/rootstock combinations achieve contrasting epigenetic configurations through different patterns of methylome remodeling, highlighting that similar physiological outcomes may arise from distinct epigenetic trajectories.

In contrast, LP/SM displayed a pronounced amplitude between control and drought conditions, with LP^C^/SM showing the highest methylation levels across contexts and LP^D^/SM the lowest, a dynamic pattern suggestive of active epigenetic remodeling under stress. This divergence was consistent across gene bodies, flanking regions, and TE‐associated sequences (Figure 3), suggesting that LP/SM appears to undergo broader epigenetic reorganization during recurrent drought exposure, whereas LP/RL maintains a more stable methylome architecture, consistent with stable epigenetic configurations following recurrent drought cycles (Neves et al. 2017; Sousa et al. 2022). Locus‐specific methylation changes were therefore investigated through DMR analysis to identify the precise genomic targets underlying these contrasting epigenetic strategies.

### 4.3 LP/RL and LP/SM Exhibit Contrasting DMR Profiles Concentrated in Regulatory Regions

Although LP/SM exhibited greater amplitude in global methylation levels between control and drought‐exposed plants (Figures 2 and 3), DMR analysis revealed that this pattern was not reflected in a higher number of locus‐specific changes: LP/RL harbored far more DMRs (8835) than LP/SM (407), indicating that the two combinations differ not only in the magnitude but also in the nature of their epigenetic responses to recurrent drought. The majority of DMRs were detected in the CHH context and concentrated in promoters and transposable elements (TEs) rather than gene bodies, suggesting that drought‐associated methylation changes are preferentially located in regulatory regions across both combinations.

Several non‐mutually exclusive mechanisms have been proposed to explain how rootstocks influence systemic epigenetic responses in grafted plants. The observations reported here are discussed in the context of these hypotheses. The predominance of CHH DMRs in these features is consistent with the potential involvement of the RNA‐directed DNA methylation (RdDM) pathway, which is known to establish de novo CHH methylation at promoters and TEs (Bai et al. 2025). Although direct evidence of RdDM activation was not assessed in this study, this pattern was particularly pronounced in LP/RL, where CHH DMRs were enriched across multiple TE superfamilies, particularly LTR/Gypsy and LTR/Copia (Figure 4b). In contrast, LP/SM showed enrichment restricted to a single superfamily (DNA/PIF Harbinger), which is consistent with a more restricted distribution of CHH‐associated DMRs in this combination rather than direct evidence of differential RdDM activity.

In LP/RL, drought‐exposed plants exhibited predominantly CHH hypomethylation across genic regions and TEs, accompanied by localized CG hypermethylation in promoters, suggesting potential remodeling of transcriptional regulation in gene‐associated regions and reinforcement of regulatory methylation at specific loci (Figure 4a). In contrast, LP/SM showed a markedly reduced number of DMRs, with promoter hypomethylation in CG, absence of significant CHG DMRs, and a tendency toward slight CHH hypermethylation in promoters, gene bodies, and exons, accompanied by hypomethylation in TEs (Figure 4a), reflecting a far more targeted epigenetic response (Figure 4b).

These contrasting DMR profiles reflect distinct epigenetic configurations associated with each scion/rootstock combination. In LP/RL, the broad CHH hypomethylation across genes and TEs may be associated with transcriptional reprogramming and TE‐associated regulatory processes, potentially contributing to the maintenance of persistent epigenetic states following recurrent drought (Lu et al. 2017; Sousa et al. 2022). The concurrent CG hypermethylation in promoters suggests a complementary mechanism of targeted gene silencing, potentially fine‐tuning the expression of specific drought‐responsive loci. In LP/SM, the more restricted DMR landscape—concentrated in fewer but potentially more functionally relevant genomic regions—may reflect a precise and targeted epigenetic strategy, consistent with the dehydration tolerance approach previously described for this rootstock (Santana‐Vieira et al. 2016; Neves et al. 2017).

Together, these findings suggest that persistent drought‐associated epigenetic states in citrus are represented by combination‐specific DMR landscapes that reflect the distinct physiological strategies of LP/RL and LP/SM. To further characterize the biological processes potentially affected by these epigenetic changes, GO and KEGG enrichment analyses were performed on genes associated with hyper‐ and hypomethylated DMRs in each combination.

Although the present study was not designed to identify the mechanisms by which rootstocks influence DNA methylation patterns in the scion, several non‐mutually exclusive mechanisms may account for the contrasting methylation landscapes observed between the two scion/rootstock combinations. Rootstocks can alter scion physiology through changes in hydraulic conductance, hormonal signaling (particularly ABA), and the long‐distance movement of signaling molecules, including mobile small RNAs capable of influencing RNA‐directed DNA methylation (RdDM). These mechanisms have been proposed in grafted plants and provide biologically plausible explanations for the contrasting CHH methylation patterns observed here. However, because neither small RNAs nor RdDM activity were directly assessed in this study, these possibilities should be regarded as hypotheses for future investigation rather than conclusions supported by the present data.

### 4.4 GO and KEGG Analyses Link Rootstock‐Specific DMR Patterns to Distinct Drought Response Pathways

To investigate the biological processes potentially associated with the rootstock‐specific DMR landscapes identified above, GO and KEGG enrichment analyses were performed separately for hypermethylated and hypomethylated DMRs in the CHH context, the most responsive to recurrent drought, for both LP^C^/RL_LP^D^/RL and LP^C^/SM_LP^D^/SM comparisons (Figure 6). The analyses revealed contrasting functional signatures between the two combinations, with no overlap in enriched GO terms between LP/RL and LP/SM, indicating that the genotype‐specific DMR landscapes identified above are associated with distinct biological processes potentially modulated by epigenetic regulation under recurrent water deficit.

In LP^C^/RL_LP^D^/RL, GO enrichment analysis revealed contrasting functional signatures between hypermethylated and hypomethylated DMR sets (Figure 6a). Hypermethylated DMRs were significantly enriched in carbon fixation pathways, a pattern consistent with the physiological behavior previously described for this combination, characterized by enhanced water uptake capacity and the maintenance of CO_2_ assimilation and growth under water deficit conditions, in line with the dehydration avoidance strategy of the “Rangpur” rootstock (Santana‐Vieira et al. 2016; Neves et al. 2017, 2018).

Hypermethylated DMRs were also enriched in receptor‐ and kinase‐mediated signaling pathways, including cell surface receptor serine/threonine kinase activity and enzyme‐linked receptor signaling. Rather than indicating suppression of stress perception, this enrichment may reflect modulation of signaling‐related loci under recurrent drought conditions. This pattern aligns with previous observations indicating that RL‐associated combinations maintain a more avoidance‐oriented physiological profile, linked to greater water acquisition capacity and more sustained carbon assimilation under stress (Neves et al. 2017; Sousa et al. 2022).

Conversely, hypomethylated DMRs were enriched in DNA repair and antioxidant‐related processes, including endo/exonuclease activity and anthocyanin metabolism (Figure 6a), suggesting that genomic stability‐related pathways remain represented among loci undergoing methylation changes in LP/RL under recurrent drought. This pattern aligns with previous observations for this rootstock, which exhibits relatively lower accumulation of antioxidant metabolites under stress conditions (Santana‐Vieira et al. 2016), suggesting that regulatory adjustments may occur at the level of gene accessibility rather than through strong induction of protective responses. Together, these patterns indicate that hyper‐ and hypomethylated DMRs in LP/RL are associated with distinct functional categories, including signaling, photosynthesis, and genome maintenance processes. This distribution is consistent with a more stable and distributed pattern of methylation change across functional pathways under recurrent drought conditions (Santana‐Vieira et al. 2016; Neves et al. 2017).

In LP/SM, hypermethylated DMRs were enriched in GO terms related to stomatal closure and negative regulation of cell death (Figure 6a), suggesting that loci associated with stomatal regulation and cellular protection may participate in the epigenetic response to recurrent drought. This pattern is consistent with the dehydration tolerance strategy of the “Sunki Maravilha” rootstock, which relies on tight control of stomatal conductance and maintenance of cell viability under water deficit (Osakabe et al. 2014; Santana‐Vieira et al. 2016; Santos et al. 2020). Notably, the simultaneous enrichment of stomatal closure in hypermethylated DMRs and stomatal movement in hypomethylated DMRs suggests coordinated regulation of stomatal‐related processes, consistent with the precise control of water loss previously described for this rootstock under recurrent drought (Santana‐Vieira et al. 2016).

Conversely, hypomethylated DMRs in LP/SM were enriched in terms related to transcriptional regulation and chromatin remodeling, including helicase activity and RNA polymerase II regulatory complexes (Figure 6a), indicating that transcription‐associated loci are prominently represented among regions undergoing methylation changes in this combination. This pattern aligns with previous findings showing that SM‐associated combinations undergo progressive physiological adjustment across recurrent drought cycles, including changes in gas exchange and stress‐related responses (Neves et al. 2017), and that these responses can be maintained and influence the performance of newly established plants following vegetative propagation (Sousa et al. 2022).

KEGG enrichment analysis corroborated these GO‐based patterns, revealing contrasting pathway‐level signatures between the two combinations (Figure 6b). In LP/RL, hypermethylated DMRs were associated with carbon fixation, signal transduction, and environmental adaptation pathways, while hypomethylated DMRs were enriched in DNA repair, nucleotide excision repair, and ubiquitin‐mediated proteolysis. In LP/SM, hypermethylated DMRs were enriched in MAPK signaling and plant hormone signal transduction, consistent with the central role of hormonal regulation in dehydration tolerance (Santana‐Vieira et al. 2016), whereas hypomethylated DMRs were associated with DNA replication, mismatch repair, and α‐linolenic acid metabolism, indicating the involvement of genome maintenance and lipid‐derived signaling pathways under recurrent drought (Arora et al. 2022).

### 4.5 Expression of Drought‐Responsive Genes Corroborates Rootstock‐Dependent Epigenetic Strategies

Although no statistically significant correlations were detected between DMR methylation levels and expression of individual candidate genes (FDR < 0.05), IGV visualization confirmed that these candidate loci overlapped promoter‐ or gene body‐associated CHH DMRs (Figure S1), and the overall expression patterns observed 1 week after the third drought–recovery cycle were broadly consistent with the epigenetic patterns identified above. This is not unexpected, as the relationship between DNA methylation and gene expression is not always direct or linear. Methylation changes may influence transcription indirectly through chromatin remodeling or in combination with other epigenetic marks such as histone modifications (Zhang et al. 2018; Kinoshita and Seki 2014). Rather than establishing causal links, the expression data provide complementary evidence of rootstock‐dependent regulatory patterns, with distinct trends emerging for genes involved in methylation maintenance, ABA and calcium signaling, and redox regulation. Accordingly, the expression patterns discussed below should be interpreted as qualitative observations that provide biological context for the methylation data, rather than as statistically supported evidence of methylation‐mediated transcriptional regulation.

Among the genes analyzed, *MET1* showed the clearest qualitative correspondence between methylation and gene expression trends. *MET1*, responsible for maintaining CG methylation and a key component of methylome regulation (Finnegan and Dennis 1993), showed a tendency toward increased expression in LP^D^/RL and decreased expression in LP^D^/SM (Figure 7a), a contrast mirrored in the methylation profiles of its promoter and gene body regions (Figure 8a,d). In LP/RL, lower CG promoter methylation was associated with higher gene body methylation, a pattern frequently associated with active transcription, whereas LP/SM showed the opposite tendency. These patterns are consistent with differences in methylation maintenance dynamics between combinations, with LP/RL displaying a more stable CG methylation profile across conditions and LP/SM exhibiting a more variable pattern. This distinction aligns with previous observations of contrasting physiological and epigenetic responses under recurrent drought (Neves et al. 2017; Sousa et al. 2022), suggesting that methylation maintenance processes may differ between scion/rootstock combinations under stress.

Genes involved in ABA signaling revealed a clear contrast between combinations. In LP/RL, the tendency toward increased expression of *PYL8* and *NF‐YC9*, both associated with ABA perception and stomatal regulation (Lim et al. 2013; Bi et al. 2017; Dittrich et al. 2019), suggests that components of ABA signaling pathways are represented under drought conditions. This pattern is consistent with previous physiological observations showing that LP/RL maintains effective stomatal regulation despite relatively low ABA accumulation under water deficit (Santana‐Vieira et al. 2016). The expression trends observed for *PYL8* and *NF‐YC9* (Figure 7f,g,i) further are consistent with this interpretation, indicating that ABA signaling in this combination is associated with efficient signal transduction rather than strong hormonal accumulation under recurrent drought conditions. In contrast, LP/SM showed a tendency toward reduced *PYR1* expression and more moderate induction of *PYL8*, alongside previously reported increases in ABA levels under drought (Santana‐Vieira et al. 2016; Neves et al. 2017). This combination of expression and physiological data is consistent with a response more strongly associated with hormonal accumulation to trigger downstream responses, in line with its dehydration tolerance profile. The gene *NFXL2*, induced in both combinations but more prominently in LP/RL, may be associated with modulation of stress‐related signaling pathways during recovery, potentially contributing to the stabilization of physiological responses after rehydration (Lisso et al. 2012).

Genes involved in calcium‐mediated stress perception revealed complementary but distinct patterns between combinations. *CPK33*, a negative regulator of stomatal closure (Li et al. 2015; Chen et al. 2019), showed a tendency toward reduced expression in both combinations after rehydration, indicating a shared state of stomatal adjustment following recurrent drought cycles. In LP/RL, the tendency toward increased *PERK4* expression, a regulator of Ca^2+^ signaling associated with ABA‐related responses (Bai et al. 2009), indicates that calcium‐mediated signaling pathways are actively represented in this combination (Figure 7e,h). This pattern is consistent with previous observations suggesting effective stomatal regulation under relatively low ABA accumulation (Santana‐Vieira et al. 2016).

In LP/SM, the pronounced induction of *CAS*, involved in extracellular Ca^2+^ detection and rapid stomatal responses, and *RPK1*, associated with ABA signaling under drought (Rahim et al. 2022), suggests a stronger representation of calcium‐ and hormone‐related signaling components. This pattern is consistent with the greater hormonal accumulation previously reported for this rootstock under water deficit (Santana‐Vieira et al. 2016; Neves et al. 2017). Together, these expression patterns suggest that calcium signaling pathways are differentially represented between combinations, aligning with their contrasting physiological responses to drought (Figure 7b,e,h).

Genes related to redox regulation and proteolysis further highlighted differences between combinations. *ORTHRUS2*, a ubiquitin E3 ligase associated with protein ubiquitination and redox homeostasis (Chen, Song, et al. 2020; Chen, Chen, et al. 2020; Kamruzzaman et al. 2022), showed relatively stable expression in LP/RL but a tendency toward increased expression in LP/SM under drought (Figure 7d). This pattern is consistent with previous observations indicating higher accumulation of reactive oxygen species in SM‐associated combinations under water deficit (Santana‐Vieira et al. 2016), suggesting a greater involvement of protein turnover and redox‐related processes in this combination. In contrast, the more stable expression of *ORTHRUS2* in LP/RL is consistent with a physiological profile characterized by lower oxidative stress levels under drought, reducing the demand for stress‐induced proteolytic activity. Similarly, *NAC029*, a transcriptional regulator associated with stress and antioxidant responses (Niu et al. 2024; Li et al. 2023), showed a tendency toward induction in both combinations, but more pronounced in LP/SM (Figure 7c). This pattern indicates a stronger representation of stress‐responsive transcriptional regulation in LP/SM, whereas the more moderate response observed in LP/RL aligns with its more stable physiological behavior under recurrent drought conditions (Santana‐Vieira et al. 2016; Neves et al. 2017).

Collectively, the expression patterns of the 11 candidate genes analyzed, spanning methylation maintenance, ABA and calcium signaling, and redox regulation, suggest differences between scion/rootstock combinations under recurrent drought. LP/RL displayed a profile characterized by relatively stable methylation patterns, representation of ABA signaling components under low hormonal accumulation, coordinated calcium‐related signaling, and moderated redox‐associated responses, aligning with the broader and more stable epigenetic patterns observed in its DMR landscape. In contrast, LP/SM exhibited a more dynamic expression profile, with stronger representation of hormone‐related signaling, calcium‐mediated responses, and proteolysis‐associated pathways, consistent with its more variable and targeted pattern of methylation change. Although direct causal links between specific DMRs and gene expression changes could not be established, the convergence between epigenomic and gene expression patterns across both combinations supports the interpretation that rootstock identity is associated with distinct regulatory configurations under recurrent water deficit. Considering that DNA methylation represents only one layer of gene regulation, the absence of significant methylation‐expression correlations does not preclude biological relevance but indicates that additional regulatory mechanisms are likely involved in shaping transcriptional responses under recurrent drought.

### 4.6 Epigenetic Memory and Rootstock Selection as Strategies for Climate‐Resilient Citrus Breeding

**FIGURE 9:**
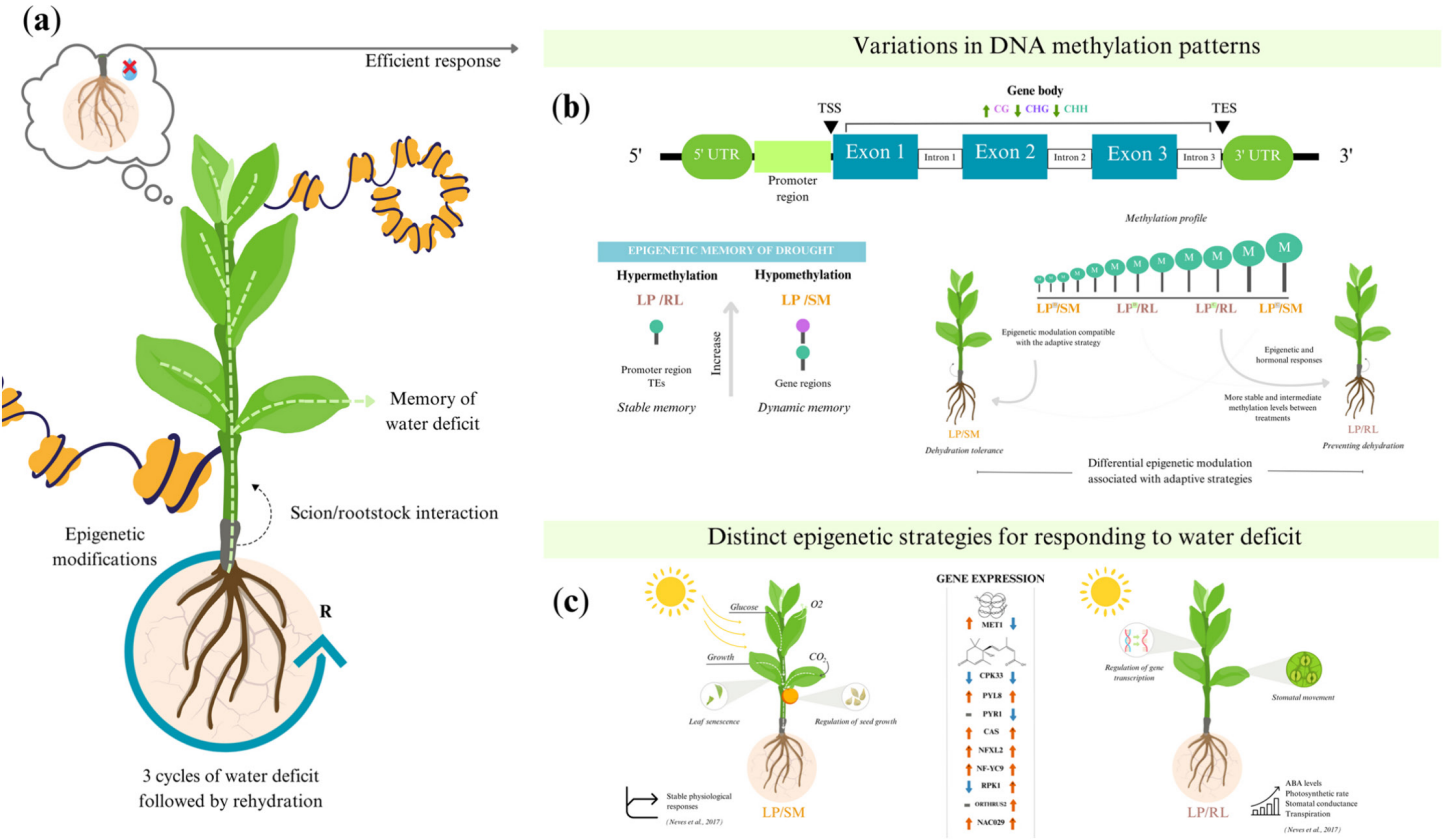
Integrative schematic representing the main findings on epigenetic regulation in citrus plants subjected to repeated cycles of water deficit followed by rehydration. The central panel highlights the epigenetic states established after three stress cycles, potentially associated with drought stress memory, with DNA methylation changes associated with scion/rootstock interaction. On the left (a), a citrus plant with a specific scion/rootstock combination is shown undergoing repeated drought and rehydration cycles, promoting an acclimation process consistent with the development of drought stress memory. Consistent with stress memory establishment, plants exposed to subsequent drought events may respond more efficiently through rootstock‐dependent changes in DNA methylation patterns (b), activating distinct epigenetic strategies (c). In panel (b), methylation profiles across gene bodies and promoter regions are presented, highlighting alterations in CG, CHG, and CHH contexts. Hypermethylation, particularly in the CHH context in promoters and TEs, is consistent with a more stable epigenetic memory in LP/RL, whereas hypomethylation in gene regions reflects a more dynamic epigenetic response in LP/SM. This differential methylation between scion/rootstock combinations is associated with contrasting stress response strategies: LP/SM shows greater dehydration tolerance with epigenetic patterns consistent with an efficient adaptive response, while LP/RL maintains more stable methylation levels associated with dehydration avoidance. In panel (c), arrows indicate tendencies in gene expression in response to water deficit compared to control conditions. Upward arrows indicate tendency toward higher expression and downward arrows indicate tendency toward lower expression. The association between methylation changes and expression patterns is shown for illustrative purposes, as direct causal links were not established in this study. Differences between scion/rootstock combinations (LP/SM and LP/RL) are shown under both control and stress conditions.

This study provides evidence that citrus scion/rootstock combinations exhibit distinct DNA methylation landscapes, gene expression patterns, and physiological responses following recurrent drought–recovery cycles (Figure 9). These differences were consistently associated with rootstock identity, with LP/RL and LP/SM displaying contrasting regulatory and physiological profiles under water deficit. The convergence between these findings and previous studies using the same rootstocks under different experimental contexts, including during stress application (Neves et al. 2017) and following vegetative transmission (Sousa et al. 2022), supports the interpretation that rootstock identity is a key factor associated with differential drought responses in citrus. Notably, while the present study focused on methylation patterns after stress recovery rather than dynamic responses during stress imposition, the consistency with observations obtained under active stress conditions (Neves et al. 2017) suggests that the methylation configurations identified here are unlikely to be random but reflect stable regulatory states associated with prior drought exposure.

From an applied perspective, the epigenetic patterns and candidate genes identified here provide a framework for exploring rootstock‐informed strategies aimed at improving drought resilience in citrus. Notably, the plants analyzed in this study were budwood mother plants, the material used for commercial propagation, highlighting the potential relevance of these findings for nursery systems. Previous work has demonstrated that stress‐associated responses can be maintained following vegetative propagation, influencing the physiological performance of newly established plants (Sousa et al. 2022). In this context, the methylation patterns observed here may be relevant for understanding how prior environmental exposure could influence the performance of propagated material. These findings suggest potential avenues for the development of propagation strategies that consider the physiological and epigenetic history of source plants, although further studies under field conditions will be required to assess their practical implications.

Future investigations should address the stability and reversibility of the methylation patterns identified here under field conditions, particularly in systems where plants experience heterogeneous and fluctuating water availability. In addition, the contribution of other epigenetic layers, including histone modifications and small RNA pathways, should be explored to provide a more comprehensive view of regulatory responses to recurrent drought in grafted citrus. Integrating these approaches with time‐resolved physiological and gene expression analyses will be important to better understand how epigenetic states are associated with functional responses across successive drought events. Such efforts will help clarify the extent to which the patterns described here are maintained under agronomic conditions and how they relate to long‐term plant performance. Together, these approaches will help establish whether the persistent methylation states identified here represent stable epigenetic signatures associated with drought history and how they contribute to long‐term adaptation in grafted citrus.

## Author Contributions

M.A.A.S. conducted the experiment, performed data analyses, prepared the figures, and wrote the manuscript. F.S.M. and I.S. processed the data, prepared the figures, and contributed to the manuscript revision. M.A.C.‐F. established and supervised the drought stress conditions for the experiment. C.F.F. facilitated the sample submission for sequencing and coordinated the associated resources. F.M. provided the bioinformatics analysis platform and RT‐qPCR facilities at CIRAD and contributed to the supervision of data analysis. A.S.G. conceived and designed the study, secured funding, participated in the experimental setup and sample collection, supervised the project, and revised the manuscript. All authors contributed to the article and approved the submitted version.

## Funding

This work was supported by Fundacão de Amparo à Pesquisa do Estado da Bahia and Brazilian National Council for Scientific and Technological Development (CNPq) (402255/2023‐2).

## Conflicts of Interest

The authors declare no conflicts of interest.

## Supporting information


**Figure S1:** IGV snapshots of representative CHH DMRs overlapping drought‐responsive candidate genes. Panels (a–c) show gene body‐associated DMRs in CAS, CAS, and CPK3, respectively, whereas panels (d–f) show promoter‐associated DMRs in CAS, NFXL2, and PERK4, respectively. For each locus, the gene annotation track is shown in orange, the CHH DMR interval in blue, and the corresponding methylation tracks for LP^C^ (green) and LP^D^ (pink) samples. Values displayed beneath the DMR track indicate the mean methylation difference between groups, whereas values beneath the methylation tracks represent the methylation fraction at individual cytosine sites.
**Table S1:** Summary statistics for WGBS analysis of citrus plant samples subjected to severe water deficit cycles.
**Table S2:** Summary of drought cycle characteristics for each scion/rootstock combination. For each cycle, the table presents the drought duration (days to reach −40 kPa), the rehydration threshold applied, and the recovery period. The −25 kPa threshold was used solely for monitoring purposes. Sampling was performed 1 week after the final rehydration of Cycle 3.
**Table S3:** Fisher's exact test results for DMR enrichment by genomic feature. Summary statistics from Fisher's exact tests evaluating enrichment or depletion of DMRs across promoters, gene bodies, and transposable elements (TEs). Columns: Context—Cytosine methylation context (CG, CHG, CHH). Comparison—Treatment/Genotype comparison (LP^C^/RL_LP^D^/RL, LP^C^/SM_LP^D^/SM, LP^C^/RL_LP^C^/SM, LP^D^/RL_LP^D^/SM). Feature—Genomic feature tested (Promoters, Gene bodies, TEs). (a–d) Counts from the 2 × 2 contingency table: (a) DMR bp overlapping feature; (b) DMR bp outside feature; (c) feature bp outside DMRs; (d) remaining genomic bp. odds_ratio: Fisher's odds ratio. fisher_p: Raw *p* value from Fisher's exact test. fisher_p_adj: Benjamini–Hochberg FDR‐adjusted *p* value. log2_OR: Log_2_‐transformed odds ratio. has_dmrs: TRUE if DMRs were detected genome‐wide for a given comparison. Testable: TRUE if test could be computed; FALSE if no DMRs were detected genome‐wide for a given comparison.
**Table S4:** DMR enrichment across transposable element (TE) superfamilies. Results of Fisher's exact tests evaluating enrichment or depletion of DMRs within individual TE superfamilies. Columns: Context: Methylation context (CG, CHG, CHH). Comparison: Treatment/Genotype comparison (LP^C^/RL_LP^D^/RL, LP^C^/SM_LP^D^/SM, LP^C^/RL_LP^C^/SM, LP^D^/RL_LP^D^/SM). Superfamily: TE superfamily. (a–d) Contingency table counts: (a) DMR bp in superfamily; (b) DMR bp outside superfamily; (c) superfamily bp outside DMRs; (d) remaining genomic bp. odds_ratio: Fisher's odds ratio. p_value: Raw *p* value. p_adj: FDR‐adjusted *p* value. Enrichment: Interpretation (Enriched, Depleted, or Not significant; based on FDR < 0.05 and OR > 1 or < 1).
**Table S5:** Summary of DMR density across genomic features and transposable element (TE) superfamilies. The table presents the number of DMRs and the total number of DMR‐overlapping base pairs normalized by feature length (DMRs per kilobase and bp per kilobase, respectively) for each methylation context (CG, CHG, and CHH). Values are reported for all treatment and genotype comparisons (LP^C^/RL_LP^D^/RL, LP^C^/SM_LP^D^/SM, LP^C^/RL_LP^C^/SM, and LP^D^/RL_LP^D^/SM). DMR density was calculated separately for promoters, gene bodies, and TE categories, including individual TE superfamilies. To assess deviations from genome‐wide expectations, binomial and Poisson tests were applied to evaluate DMR coverage and count rates, respectively. Columns include: context (methylation context), comparison (treatment/genotype comparison), feature (promoter, gene body, or TE superfamily), feature_length_kb (feature length in kilobases), DMR_count (number of overlapping DMRs), DMR_bp (total number of DMR base pairs overlapping the feature), DMRs_per_kb (number of DMRs per kilobase), bp_per_kb (number of overlapping DMR base pairs per kilobase), p_value (raw *p* value), and p_adj (FDR‐adjusted *p* value).
**Table S6:** Statistical summary of RT–qPCR gene expression analyses. For each candidate gene, pairwise comparisons were performed between (i) control and drought treatments within each graft combination (LP^C^/RL_LP^D^/RL; LP^C^/SM_LP^D^/SM) and (ii) the two graft combinations under the same treatment (LP^C^/RL_LP^C^/SM; LP^D^/RL_LP^D^/SM). Expression values were analyzed as log_2_ fold changes (log_2_FC). The Shapiro–Wilk test was used to assess normality of replicate distributions; if both groups were normally distributed (*p* > 0.05), Welch's *t*‐test was applied, otherwise a Wilcoxon rank‐sum test was used. Reported values include mean log_2_FC per group, mean differences, *p* values, adjusted *p* values (Benjamini–Hochberg correction), and Shapiro–Wilk *p* values for each group. No statistically significant differences were observed after multiple testing correction (FDR < 0.05).

## Data Availability

The whole‐genome bisulfite sequencing data generated in this study have been deposited in the NCBI Sequence Read Archive (SRA) under accession number PRJNA1282485. Additional datasets supporting the findings of this study are available from the corresponding author upon request.
