# Supplementary material for: Persistent Epigenetic Reprogramming Is Associated With Contrasting Drought‐Response Strategies in Citrus Scion–Rootstock Systems
Source: Physiol Plant. 2026 Sep 22;178(5):e71113. doi: 10.1111/ppl.71113 (PMC13597148; doi:10.1111/ppl.71113)
Supplement: Supplementary file 1 [file PPL-178-e71113-s001.zip › ppl71113-sup-0001-Supinfo.docx]

**Persistent epigenetic reprogramming is associated with contrasting drought-response strategies in citrus scion–rootstock systems**

Monique Ayala Araújo da Silva¹; Fredson dos Santos Menezes¹; Iris Sammarco²; Lucas da Silva Costa¹; Claudia Fortes Ferreira³; Walter dos Santos Soares Filho^3^; Fabienne Micheli¹^,4,5^; Maurício Antônio Coelho-Filho³; Abelmon da Silva Gesteira¹^,^³.

**Supplementary Material**

**Supplementary Figure S1.** IGV snapshots of representative CHH DMRs overlapping drought-responsive candidate genes. Panels (a-c) show gene body-associated DMRs in CAS, CAS, and CPK3, respectively, whereas panels (d-f) show promoter-associated DMRs in CAS, NFXL2, and PERK4, respectively. For each locus, the gene annotation track is shown in orange, the CHH DMR interval in blue, and the corresponding methylation tracks for LP^C^ (green) and LP^D^ (pink) samples. Values displayed beneath the DMR track indicate the mean methylation difference between groups, whereas values beneath the methylation tracks represent the methylation fraction at individual cytosine sites.

|  |  |  |  | **Mapped Results** | | | | |
| --- | --- | --- | --- | --- | --- | --- | --- | --- |
| **Sample_ID** | **Filtered Reads** | | | **Unique Alignments** | | **Multiple mapped** | |  |
|  | **Total Reads** | **Bissulfite Conversion rate (%)** | **Duplication Rate (%)** | **Total Reads** | **Unique Alignments Rate(%)** | **Multiple Alignments** | **Multiple Alignments Rate(%)** | **No Alignments Reads Rate (%)** |
| **LC1** | 46455764 | 99.5 | 15.26 | 22806635 | 49.1 | 4578866 | 9.86 | 41.1 |
| **LC2** | 41625092 | 99.47 | 18.91 | 20581298 | 49.4 | 3942964 | 9.47 | 41.1 |
| **LC3** | 47547531 | 99.48 | 15.63 | 22957886 | 48.3 | 4849821 | 10.2 | 41.5 |
| **LD1** | 38294061 | 99.49 | 13.62 | 19575150 | 51.1 | 4782418 | 12.5 | 36.4 |
| **LD2** | 46071570 | 99.47 | 15.04 | 21559321 | 46.8 | 4500842 | 9.8 | 43.4 |
| **LD3** | 40920601 | 99.46 | 15.05 | 20513752 | 50.1 | 4009603 | 9.8 | 40.1 |
| **SC1** | 40403647 | 99.45 | 15.67 | 20743303 | 51.3 | 4892436 | 12.1 | 36.6 |
| **SC2** | 48525864 | 99.46 | 14.76 | 23700898 | 48.8 | 4695161 | 9.7 | 41.5 |
| **SC3** | 41550397 | 99.46 | 16.41 | 21206356 | 51.0 | 4341069 | 10.4 | 38.5 |
| **SD1** | 38149249 | 99.5 | 15.05 | 19124557 | 50.1 | 4430720 | 11.6 | 38.3 |
| **SD2** | 51931342 | 99.46 | 6.81 | 23278578 | 44.8 | 5283061 | 10.2 | 45.0 |
| **SD3** | 60285536 | 99.48 | 9.14 | 23780024 | 39.4 | 5711215 | 9.5 | 51.1 |
| **Average** | 45146721 | 99.47 | 14.28 | 21652313 | 48.35 | 4668181 | 10.42 | 41.22 |
| **Total** | 541760654 | 1193.68 | 171.35 | 259827758 | 580.2 | 56018176 | 125.05 | 494.6 |

**Supplementary Table S1.** Summary statistics for WGBS analysis of citrus plant samples subjected to severe water deficit cycles.

**Supplementary Table S2.** Summary of drought cycle characteristics for each scion/rootstock combination. For each cycle, the table presents the drought duration (days to reach –40 kPa), the rehydration threshold applied, and the recovery period. The –25 kPa threshold was used solely for monitoring purposes. Sampling was performed one week after the final rehydration of cycle 3.

| **Cycle** | **LPD/RL duration (days)** | **LPD/SM duration (days)** | **Rehydration threshold** | **Recovery period** |
| --- | --- | --- | --- | --- |
| 1 | 17 | 14 | −40 kPa | 7 days |
| 2 | 24 | 29 | −40 kPa | 7 days |
| 3 | 30 | 27 | −40 kPa | 7 days |
| Sampling | — | — | — | 1 week after cycle 3 |

**Supplementary Table S3.** Fisher’s exact test results for DMR enrichment by genomic feature. Summary statistics from Fisher’s exact tests evaluating enrichment or depletion of DMRs across promoters, gene bodies, and transposable elements (TEs).
Columns*:* Context – Cytosine methylation context (CG, CHG, CHH). Comparison – Treatment/Genotype comparison (LP^C^/RL_LP^D^/RL, LP^C^/SM_LP^D^/SM, LP^C^/RL_LP^C^/SM, LP^D^/RL_LP^D^/SM). Feature – Genomic feature tested (Promoters, Gene bodies, TEs). a - d: Counts from the 2×2 contingency table: (a) DMR bp overlapping feature; (b) DMR bp outside feature; (c) feature bp outside DMRs; (d) remaining genomic bp. odds_ratio: Fisher’s odds ratio. fisher_p: Raw p-value from Fisher’s exact test. fisher_p_adj: Benjamini–Hochberg FDR-adjusted p-value. log2_OR: Log₂-transformed odds ratio. has_dmrs: TRUE if DMRs were detected genome-wide for a given comparison. testable: TRUE if test could be computed; FALSE if no DMRs were detected genome-wide for a given comparison.

Context,Comparison,Feature,a,b,c,d,odds_ratio,fisher_p,testable,fisher_p_adj,log2_OR,has_dmrs,sig,category

CHG,LPC/RL_LPD/RL,Promoters,0,1054,45607783,254169709,0,4.140411047788874e-76,TRUE,7.097847510495212e-76,#NAME?,FALSE,TRUE,sig

CHG,LPC/RL_LPD/RL,Gene bodies,20,1034,33391298,266386194,0.15430778512459986,1.7620530676829e-30,TRUE,2.4165299213936913e-30,-2.696117244,TRUE,TRUE,sig

CHG,LPC/RL_LPD/RL,TEs,0,1054,76264427,223513065,0,6.10454185831055e-135,TRUE,1.22090837166211e-134,#NAME?,FALSE,TRUE,sig

CHG,LPC/RL_LPC/SM,Promoters,704,5191,45607079,254165572,0.7557992413007686,5.745550771520008e-13,TRUE,6.56634373888001e-13,-0.403925025,TRUE,TRUE,sig

CHG,LPC/RL_LPC/SM,Gene bodies,1295,4600,33390023,266382628,2.246007288956573,1.4335539054842489e-124,TRUE,2.6465610562786133e-124,1.1673626097173377,TRUE,TRUE,sig

CHG,LPC/RL_LPC/SM,TEs,749,5146,76263678,223508973,0.42654343137232725,1.379086603367929e-128,TRUE,2.647846278466424e-128,-1.229235448,TRUE,TRUE,sig

CHG,LPD/RL_LPD/SM,Promoters,0,0,45607783,254170763,NA,NA,FALSE,NA,NA,FALSE,FALSE,untestable

CHG,LPD/RL_LPD/SM,Gene bodies,0,0,33391318,266387228,NA,NA,FALSE,NA,NA,FALSE,FALSE,untestable

CHG,LPD/RL_LPD/SM,TEs,0,0,76264427,223514119,NA,NA,FALSE,NA,NA,FALSE,FALSE,untestable

CHG,LPC/SM_LPD/SM,Promoters,0,0,45607783,254170763,NA,NA,FALSE,NA,NA,FALSE,FALSE,untestable

CHG,LPC/SM_LPD/SM,Gene bodies,0,0,33391318,266387228,NA,NA,FALSE,NA,NA,FALSE,FALSE,untestable

CHG,LPC/SM_LPD/SM,TEs,0,0,76264427,223514119,NA,NA,FALSE,NA,NA,FALSE,FALSE,untestable

CHH,LPC/RL_LPD/RL,Promoters,146474,644053,45461309,253526710,1.2682953846131788,0,TRUE,0,0.34289078671376294,TRUE,TRUE,sig

CHH,LPC/RL_LPD/RL,Gene bodies,2508,788019,33388810,265599209,0.025327567,0,TRUE,0,-5.303147721,TRUE,TRUE,sig

CHH,LPC/RL_LPD/RL,TEs,211545,578982,76052882,222935137,1.0710344067516573,1.4725793737154337e-158,TRUE,3.3658957113495626e-158,0.099004827,TRUE,TRUE,sig

CHH,LPC/RL_LPC/SM,Promoters,20134,63745,45587649,254107018,1.7605670949010104,0,TRUE,0,0.8160402091019051,TRUE,TRUE,sig

CHH,LPC/RL_LPC/SM,Gene bodies,714,83165,33390604,266304063,0.068471704,0,TRUE,0,-3.868348276,TRUE,TRUE,sig

CHH,LPC/RL_LPC/SM,TEs,13226,70653,76251201,223443466,0.5485832683379951,0,TRUE,0,-0.866217474,TRUE,TRUE,sig

CHH,LPD/RL_LPD/SM,Promoters,20783,86642,45587000,254084121,1.3369516040505725,3.397651182414508e-291,TRUE,9.060403153105353e-291,0.4189472426489685,TRUE,TRUE,sig

CHH,LPD/RL_LPD/SM,Gene bodies,556,106869,33390762,266280359,0.041515126,0,TRUE,0,-4.590219098,TRUE,TRUE,sig

CHH,LPD/RL_LPD/SM,TEs,20957,86468,76243470,223427651,0.7102583229329691,0,TRUE,0,-0.493584263,TRUE,TRUE,sig

CHH,LPC/SM_LPD/SM,Promoters,4959,17312,45602824,254153451,1.5964267350817856,1.1752777950369659e-169,TRUE,2.820666708088718e-169,0.6748463447278837,TRUE,TRUE,sig

CHH,LPC/SM_LPD/SM,Gene bodies,275,21996,33391043,266365232,0.099732459,0,TRUE,0,-3.325793072,TRUE,TRUE,sig

CHH,LPC/SM_LPD/SM,TEs,2774,19497,76261653,223494622,0.41694046257101924,0,TRUE,0,-1.262086708,TRUE,TRUE,sig

CG,LPC/RL_LPD/RL,Promoters,1115,1240,45606668,254169523,5.011428084,1.2969924830555008e-295,TRUE,3.6620964227449436e-295,2.325221780163329,TRUE,TRUE,sig

CG,LPC/RL_LPD/RL,Gene bodies,533,1822,33390785,266385406,2.333842919400025,1.2200689143166715e-56,TRUE,1.8891389641032332e-56,1.2227074629180952,TRUE,TRUE,sig

CG,LPC/RL_LPD/RL,TEs,439,1916,76263988,223512203,0.6715250042951327,6.0140341680203075e-15,TRUE,7.21684100162437e-15,-0.574486975,TRUE,TRUE,sig

CG,LPC/RL_LPC/SM,Promoters,1099,1131,45606684,254169632,5.415487583578756,5.3197719573827e-311,TRUE,1.5959315872148e-310,2.4370912367704944,TRUE,TRUE,sig

CG,LPC/RL_LPC/SM,Gene bodies,186,2044,33391132,266385184,0.7259581339858076,1.5984528019257442e-5,TRUE,1.7437666930099027e-5,-0.462041745,TRUE,TRUE,sig

CG,LPC/RL_LPC/SM,TEs,123,2107,76264304,223512012,0.17108617987493024,5.917537430429901e-137,TRUE,1.2349643333071096e-136,-2.547204869,TRUE,TRUE,sig

CG,LPD/RL_LPD/SM,Promoters,254,0,45607529,254170763,Inf,1.94221560187153e-208,TRUE,4.906649941570181e-208,Inf,TRUE,TRUE,sig

CG,LPD/RL_LPD/SM,Gene bodies,0,254,33391318,266386974,0,1.452353495701358e-13,TRUE,1.7003162876503702e-13,#NAME?,FALSE,TRUE,sig

CG,LPD/RL_LPD/SM,TEs,0,254,76264427,223513865,0,5.5153990490874964e-33,TRUE,8.02239861685454e-33,#NAME?,FALSE,TRUE,sig

CG,LPC/SM_LPD/SM,Promoters,957,233,45606826,254170530,22.88640026349816,0,TRUE,0,4.516418658,TRUE,TRUE,sig

CG,LPC/SM_LPD/SM,Gene bodies,0,1190,33391318,266386038,0,2.1244791021555307e-61,TRUE,3.399166563448849e-61,#NAME?,FALSE,TRUE,sig

CG,LPC/SM_LPD/SM,TEs,0,1190,76264427,223512929,0,2.872216214319156e-152,TRUE,6.266653558514522e-152,#NAME?,FALSE,TRUE,sig

**Supplementary Table S4.** DMR enrichment across transposable element (TE) superfamilies. Results of Fisher’s exact tests evaluating enrichment or depletion of DMRs within individual TE superfamilies. Columns: Context: Methylation context (CG, CHG, CHH). Comparison: Treatment/Genotype comparison (LP^C^/RL_LP^D^/RL, LP^C^/SM_LP^D^/SM, LP^C^/RL_LP^C^/SM, LP^D^/RL_LP^D^/SM). Superfamily: TE superfamily. a-d: Contingency table counts: (a) DMR bp in superfamily; (b) DMR bp outside superfamily; (c) superfamily bp outside DMRs; (d) remaining genomic bp. odds_ratio: Fisher’s odds ratio. p_value: Raw p-value. p_adj: FDR-adjusted p-value. Enrichment: Interpretation (Enriched, Depleted, or Not significant; based on FDR < 0.05 and OR > 1 or < 1).

Context,Comparison,Feature,Feature_bp,DMR_bp_overlap,DMR_region_count,DMRs_per_kb,DMR_bp_per_kb,binom_p,poisson_p,rate_feat_per_bp,rate_rest_per_bp,rate_ratio,total_DMR_bp,total_DMR_regions,testable,binom_p_adj,poisson_p_adj,has_dmrs,sig,category,log2_RR

CHG,LCLD,Promoters,45607783,0,0,0,0,4.14238087309488e-76,0.6043722777429552,0,2.7540539743353567e-8,0,1054,7,TRUE,7.101224353876937e-76,0.7282734005573278,FALSE,TRUE,sig,#NAME?

CHG,LCLD,Gene bodies,33391318,20,1,2.994790442234116e-5,5.989580884468232e-4,1.7624476668469173e-30,0.5624880207657166,2.994790442234116e-8,2.252360237030583e-8,1.3296232072460674,1054,7,TRUE,2.4170710859614866e-30,0.7282734005573278,TRUE,TRUE,sig,0.41101746824272417

CHG,LCLD,TEs,76264427,0,0,0,0,6.10966021572987e-135,0.20278584562649726,0,3.131793209000815e-8,0,1054,7,TRUE,1.221932043145974e-134,0.37437386884891805,FALSE,TRUE,sig,#NAME?

CHG,LCSC,Promoters,45607783,704,3,6.577824666460985e-5,0.015435961883961779,5.748525100208623e-13,0.735216212,6.577824666460986e-8,5.5081079486707134e-8,1.194207653110435,5895,17,TRUE,6.569742971666997e-13,0.8266404832584869,TRUE,TRUE,sig,0.25605371940431576

CHG,LCSC,Gene bodies,33391318,1295,7,2.0963533095638812e-4,0.038782536226931806,1.442235966899743e-124,0.001496755,2.0963533095638812e-7,3.753933728384305e-8,5.584417470433483,5895,17,TRUE,2.6625894773533716e-124,0.004226131,TRUE,TRUE,sig,2.481406796134122

CHG,LCSC,TEs,76264427,749,3,3.933681951088415e-5,0.009821093,1.3868189795241273e-128,0.5859779515878976,3.933681951088415e-8,6.26358641800163e-8,0.6280238969455201,5895,17,TRUE,2.662692440686324e-128,0.7282734005573278,TRUE,TRUE,sig,-0.671108639

CHG,LDSD,Promoters,45607783,0,0,0,0,NA,NA,NA,NA,NA,0,0,FALSE,NA,NA,FALSE,FALSE,untestable,NA

CHG,LDSD,Gene bodies,33391318,0,0,0,0,NA,NA,NA,NA,NA,0,0,FALSE,NA,NA,FALSE,FALSE,untestable,NA

CHG,LDSD,TEs,76264427,0,0,0,0,NA,NA,NA,NA,NA,0,0,FALSE,NA,NA,FALSE,FALSE,untestable,NA

CHG,SCSD,Promoters,45607783,0,0,0,0,NA,NA,NA,NA,NA,0,0,FALSE,NA,NA,FALSE,FALSE,untestable,NA

CHG,SCSD,Gene bodies,33391318,0,0,0,0,NA,NA,NA,NA,NA,0,0,FALSE,NA,NA,FALSE,FALSE,untestable,NA

CHG,SCSD,TEs,76264427,0,0,0,0,NA,NA,NA,NA,NA,0,0,FALSE,NA,NA,FALSE,FALSE,untestable,NA

CHH,LCLD,Promoters,45607783,146474,1805,0.039576578409873595,3.2116009673173544,2e-323,3.723147124751629e-41,3.9576578409873595e-5,2.7383165230534403e-5,1.4452886682998418,790527,8765,TRUE,6.4e-323,3.5742212397615635e-40,TRUE,TRUE,sig,0.5313576717792066

CHH,LCLD,Gene bodies,33391318,2508,71,0.002126301,0.075109344,2e-323,5e-324,2.1263012139862224e-6,3.263669983457315e-5,0.06515062,790527,8765,TRUE,6.4e-323,2.37e-322,TRUE,TRUE,sig,-3.940077292

CHH,LCLD,TEs,76264427,211545,2272,0.029791085,2.773835827809996,3.9414777304321785e-158,0.3029817419249426,2.9791084642909597e-5,2.9049619008631844e-5,1.025524108734694,790527,8765,TRUE,9.00909195527355e-158,0.5193972718713301,TRUE,TRUE,sig,0.036361408

CHH,LCSC,Promoters,45607783,20134,268,0.00587619,0.4414597394484182,5e-324,2.062307048190199e-16,5.876190035371814e-6,3.1828995217675765e-6,1.8461751604739813,83879,1077,TRUE,3.5e-323,1.0998970923681061e-15,TRUE,TRUE,sig,0.8845394387685587

CHH,LCSC,Gene bodies,33391318,714,20,5.989580884468232e-4,0.021382804,5e-324,2.1256736107807547e-31,5.989580884468232e-7,3.96790795090221e-6,0.15095060063342297,83879,1077,TRUE,3.5e-323,1.7005388886246038e-30,TRUE,TRUE,sig,-2.727851598

CHH,LCSC,TEs,76264427,13226,199,0.002609342,0.17342292495031794,1e-323,6.64339596992396e-8,2.6093423608886486e-6,3.928163482146736e-6,0.6642652152202806,83879,1077,TRUE,3.5e-323,2.452946204279616e-7,TRUE,TRUE,sig,-0.590168726

CHH,LDSD,Promoters,45607783,20783,240,0.00526226,0.4556897668101955,4.318094607935099e-291,4.3542193153978205e-7,5.262259733168788e-6,3.599941980738359e-6,1.4617623732062157,107425,1155,TRUE,1.1514918954493596e-290,1.3062657946193461e-6,TRUE,TRUE,sig,0.5477088031778775

CHH,LDSD,Gene bodies,33391318,556,10,2.994790442234116e-4,0.016651034858821686,5e-324,1.1495677874670493e-44,2.994790442234116e-7,4.29825411900003e-6,0.069674579,107425,1155,TRUE,3.5e-323,1.839308459947279e-43,TRUE,TRUE,sig,-3.843223819

CHH,LDSD,TEs,76264427,20957,220,0.0028847,0.2747939088298664,1e-323,3.189803865796092e-7,2.8847000974648376e-6,4.1831809291653745e-6,0.6895948672342965,107425,1155,TRUE,3.5e-323,1.0207372370547496e-6,TRUE,TRUE,sig,-0.536179059

CHH,SCSD,Promoters,45607783,4959,104,0.002280313,0.10873144173660008,1.2105800377025387e-169,3.058085994193654e-8,2.2803125510398083e-6,1.172440120502766e-6,1.9449287952223644,22271,402,TRUE,2.905392090486093e-169,1.2232343976774617e-7,TRUE,TRUE,sig,0.9597173384196945

CHH,SCSD,Gene bodies,33391318,275,8,2.3958323537872928e-4,0.008235674,5e-324,5.5901454168960576e-12,2.395832353787293e-7,1.4790498889834163e-6,0.16198455316703356,22271,402,TRUE,3.5e-323,2.4393361819182795e-11,TRUE,TRUE,sig,-2.62607185

CHH,SCSD,TEs,76264427,2774,58,7.605118438770936e-4,0.036373446,5e-324,1.1237175444017079e-7,7.605118438770936e-7,1.539052662708972e-6,0.4941428336431805,22271,402,TRUE,3.5e-323,3.852745866520141e-7,TRUE,TRUE,sig,-1.016999977

CG,LCLD,Promoters,45607783,1115,5,1.0963041110768309e-4,0.024447581677013327,1.3066235907238127e-295,0.025323349,1.0963041110768309e-7,2.7540539743353567e-8,3.9806921770347836,2355,12,TRUE,3.689290138514295e-295,0.055250944291017096,TRUE,TRUE,sig,1.9930193135705219

CG,LCLD,Gene bodies,33391318,533,4,1.1979161768936464e-4,0.015962233,1.2214913384167848e-56,0.036534975906762135,1.1979161768936465e-7,3.003146982707444e-8,3.988869621738202,2355,12,TRUE,1.8913414272259894e-56,0.076246906,TRUE,TRUE,sig,1.9959799689638802

CG,LCLD,TEs,76264427,439,3,3.933681951088415e-5,0.005756288,6.0154907774560326e-15,1,3.933681951088415e-8,4.026591268715333e-8,0.9769260619152537,2355,12,TRUE,7.21858893294724e-15,1,TRUE,TRUE,sig,-0.033678718

CG,LCSC,Promoters,45607783,1099,4,8.770432888614647e-5,0.024096764,5.3595908587146e-311,0.072537039,8.770432888614647e-8,2.7540539743353567e-8,3.184553741627827,2230,11,TRUE,1.6078772576144e-310,0.14507407837521008,TRUE,TRUE,sig,1.6710912186831597

CG,LCSC,Gene bodies,33391318,186,2,5.989580884468232e-5,0.00557031,1.5985690801727053e-5,0.3510578461201734,5.989580884468233e-8,3.378540355545875e-8,1.7728309429947562,2230,11,TRUE,1.7438935420065874e-5,0.5435734391538168,TRUE,TRUE,sig,0.8260549675215677

CG,LCSC,TEs,76264427,123,2,2.6224546340589436e-5,0.00161281,5.929666079693125e-137,0.7405320995857279,2.6224546340589433e-8,4.026591268715333e-8,0.6512840412768357,2230,11,TRUE,1.2374955296750869e-136,0.8266404832584869,TRUE,TRUE,sig,-0.618641219

CG,LDSD,Promoters,45607783,254,2,4.385216444307323e-5,0.005569225,1.943376083859304e-208,0.023146047,4.3852164443073234e-8,0,NA,254,2,TRUE,4.909581685539295e-208,0.05290525,TRUE,TRUE,sig,NA

CG,LDSD,Gene bodies,33391318,0,0,0,0,1.4523824501351527e-13,1,0,7.50786745676861e-9,0,254,2,TRUE,1.7003501855240812e-13,1,FALSE,TRUE,sig,#NAME?

CG,LDSD,TEs,76264427,0,0,0,0,5.515651234338676e-33,1,0,8.947980597145185e-9,0,254,2,TRUE,8.022765431765348e-33,1,FALSE,TRUE,sig,#NAME?

CG,SCSD,Promoters,45607783,957,4,8.770432888614647e-5,0.020983260686010544,0,0.002352672,8.770432888614647e-8,3.934362820479081e-9,22.29187619139479,1190,5,TRUE,0,0.006273791,TRUE,TRUE,sig,4.478446140740764

CG,SCSD,Gene bodies,33391318,0,0,0,0,2.125636016429852e-61,1,0,1.8769668641921526e-8,0,1190,5,TRUE,3.4010176262877634e-61,1,FALSE,TRUE,sig,#NAME?

CG,SCSD,TEs,76264427,0,0,0,0,2.87533208900541e-152,0.3386343493226832,0,2.2369951492862964e-8,0,1190,5,TRUE,6.2734518305572575e-152,0.5418149589162932,FALSE,TRUE,sig,#NAME?

**Supplementary Table S5.** Summary of DMR density across genomic features and transposable element (TE) superfamilies. The table presents the number of DMRs and the total number of DMR-overlapping base pairs normalized by feature length (DMRs per kilobase and bp per kilobase, respectively) for each methylation context (CG, CHG, and CHH). Values are reported for all treatment and genotype comparisons (LP^C^/RL_LP^D^/RL, LP^C^/SM_LP^D^/SM, LP^C^/RL_LP^C^/SM, and LP^D^/RL_LP^D^/SM). DMR density was calculated separately for promoters, gene bodies, and TE categories, including individual TE superfamilies. To assess deviations from genome-wide expectations, binomial and Poisson tests were applied to evaluate DMR coverage and count rates, respectively. Columns include: context (methylation context), comparison (treatment/genotype comparison), feature (promoter, gene body, or TE superfamily), feature_length_kb (feature length in kilobases), DMR_count (number of overlapping DMRs), DMR_bp (total number of DMR base pairs overlapping the feature), DMRs_per_kb (number of DMRs per kilobase), bp_per_kb (number of overlapping DMR base pairs per kilobase), p_value (raw p-value), and p_adj (FDR-adjusted p-value).

Gene,"Comparison","Mean_Group1","Mean_Group2","Mean_Diff","test_used","p_value","adj_pvalue","shapiro_p_group1","shapiro_p_group2"

RPK1,"LPC/RL_LPD/RL",-3.33333305402306e-10,-1.69944444466667,-1.69944444433333,"Wilcoxon",0.382733088885226,0.509166788522938,0.933768203034558,0.0126498075220575

PYR1,"LPC/RL_LPD/RL",1.84811295318258e-17,-1.32166666666667,-1.32166666666667,"t-test (Welch)",0.286898498095164,0.450840497006686,0.782725957498276,0.188486715728799

DNMT1,"LPC/RL_LPD/RL",-9.25185853854297e-18,0.403125083333333,0.403125083333333,"t-test (Welch)",0.113720051274935,0.364102342271721,0.632738290951838,0.999999999999993

CAS,"LPC/RL_LPD/RL",9.25185853854297e-18,0.184513972333333,0.184513972333333,"t-test (Welch)",0.416591008791495,0.509166788522938,0.377908352614426,0.999999999999993

PERK4,"LPC/RL_LPD/RL",0,0.523402861,0.523402861,"t-test (Welch)",0.160095317825561,0.364102342271721,0.147950271735577,0.128686973427825

NFY-C9,"LPC/RL_LPD/RL",3.33333342418775e-10,0.471736194333333,0.471736194,"t-test (Welch)",0.181263541003179,0.364102342271721,0.291803210212532,0.639930056839016

NFXL2,"LPC/RL_LPD/RL",-9.26089355664702e-18,0.476736194666667,0.476736194666667,"t-test (Welch)",0.198601277602757,0.364102342271721,0.630883214455525,0.369273223170959

PYL8,"LPC/RL_LPD/RL",0,0.726459166666667,0.726459166666667,"t-test (Welch)",0.047716365168262,0.364102342271721,0.110587133251199,0.999999999999993

CPK33,"LPC/RL_LPD/RL",0,-0.132152694333333,-0.132152694333333,"t-test (Welch)",0.735734013447054,0.735734013447054,0.633458104360521,0.248708178038531

ORTHRUS2,"LPC/RL_LPD/RL",3.33333342418775e-10,0.0845139723333333,0.084513972,"t-test (Welch)",0.693244925103035,0.735734013447054,0.457463174092239,0.999999999999993

NAC029,"LPC/RL_LPD/RL",3.33333337783811e-10,0.389513972333333,0.389513972,"t-test (Welch)",0.166861033693405,0.364102342271721,0.443895868521609,0.999999999999993

RPK1,"LPC/SM_LPD/SM",-3.33333323896988e-10,0.365555555666667,0.365555556,"t-test (Welch)",0.582175598084011,0.650670896041026,0.393739912479266,0.208150200807706

PYR1,"LPC/SM_LPD/SM",-1.84675770046698e-17,-0.982222222,-0.982222222,"t-test (Welch)",0.379883324236705,0.596959509514822,0.486581937697605,0.308584742089423

DNMT1,"LPC/SM_LPD/SM",-4.27121445850664e-16,-0.568819436333333,-0.568819436333333,"t-test (Welch)",0.277002704473142,0.596959509514822,0.999999999999993,0.286046074770879

CAS,"LPC/SM_LPD/SM",-7.47485117783923e-16,1.04041666666667,1.04041666666667,"t-test (Welch)",0.0990585428543011,0.29097612718446,0.999999959752718,0.586039229564376

PERK4,"LPC/SM_LPD/SM",4.27123139916559e-16,0.140208341666667,0.140208341666666,"t-test (Welch)",0.532449905455767,0.650670896041026,0.999999999999993,0.765814006148416

NFY-C9,"LPC/SM_LPD/SM",1.17458849359838e-15,-0.203124991666667,-0.203124991666668,"t-test (Welch)",0.591518996400933,0.650670896041026,0.999999999999993,0.797927410127187

NFXL2,"LPC/SM_LPD/SM",4.27125963359716e-16,0.203541675,0.203541675,"t-test (Welch)",0.377801202479121,0.596959509514822,0.999999999999993,0.757105305000766

PYL8,"LPC/SM_LPD/SM",-4.27121445850664e-16,0.3375,0.3375,"t-test (Welch)",0.105809500794349,0.29097612718446,0.999999959752718,0.482164962724776

CPK33,"LPC/SM_LPD/SM",1.17458849359838e-15,-0.0611805473333334,-0.0611805473333345,"t-test (Welch)",0.899259921438865,0.899259921438865,0.999999999999993,0.796468852828161

ORTHRUS2,"LPC/SM_LPD/SM",0,1.14375,1.14375,"Wilcoxon",0.0808555983700523,0.29097612718446,0.999999999999993,0.0196896200936435

NAC029,"LPC/SM_LPD/SM",-8.54242891701329e-16,1.28041666666667,1.28041666666667,"t-test (Welch)",0.0187004551700462,0.205705006870508,0.999999999999993,0.287726227486462

RPK1,"LPC/RL_LPC/SM",-3.33333305402306e-10,-3.33333323896988e-10,-1.84946820762148e-17,"t-test (Welch)",1,1,0.933768203034558,0.393739912479266

PYR1,"LPC/RL_LPC/SM",1.84811295318258e-17,-1.84675770046698e-17,-3.69487065364956e-17,"t-test (Welch)",1,1,0.782725957498276,0.486581937697605

DNMT1,"LPC/RL_LPC/SM",-9.25185853854297e-18,-4.27121445850664e-16,-4.17869587312121e-16,"t-test (Welch)",0.999999999999999,1,0.632738290951838,0.999999999999993

CAS,"LPC/RL_LPC/SM",9.25185853854297e-18,-7.47485117783923e-16,-7.56736976322466e-16,"t-test (Welch)",0.999999999999999,1,0.377908352614426,0.999999959752718

PERK4,"LPC/RL_LPC/SM",0,4.27123139916559e-16,4.27123139916559e-16,"t-test (Welch)",0.999999999999999,1,0.147950271735577,0.999999999999993

NFY-C9,"LPC/RL_LPC/SM",3.33333342418775e-10,1.17458849359838e-15,-3.33332167830281e-10,"t-test (Welch)",0.999999999313065,1,0.291803210212532,0.999999999999993

NFXL2,"LPC/RL_LPC/SM",-9.26089355664702e-18,4.27125963359716e-16,4.36386856916363e-16,"t-test (Welch)",0.999999999999999,1,0.630883214455525,0.999999999999993

PYL8,"LPC/RL_LPC/SM",0,-4.27121445850664e-16,-4.27121445850664e-16,"t-test (Welch)",0.999999999999998,1,0.110587133251199,0.999999959752718

CPK33,"LPC/RL_LPC/SM",0,1.17458849359838e-15,1.17458849359838e-15,"t-test (Welch)",0.999999999999998,1,0.633458104360521,0.999999999999993

ORTHRUS2,"LPC/RL_LPC/SM",3.33333342418775e-10,0,-3.33333342418775e-10,"t-test (Welch)",0.999999998853363,1,0.457463174092239,0.999999999999993

NAC029,"LPC/RL_LPC/SM",3.33333337783811e-10,-8.54242891701329e-16,-3.33334192026702e-10,"t-test (Welch)",0.999999999207271,1,0.443895868521609,0.999999999999993

RPK1,"LPD/RL_LPD/SM",-1.69944444466667,0.365555555666667,2.06500000033333,"Wilcoxon",0.0808555983700523,0.177882316414115,0.0126498075220575,0.208150200807706

PYR1,"LPD/RL_LPD/SM",-1.32166666666667,-0.982222222,0.339444444666667,"t-test (Welch)",0.493985552965703,0.543384108262274,0.188486715728799,0.308584742089423

DNMT1,"LPD/RL_LPD/SM",0.403125083333333,-0.568819436333333,-0.971944519666667,"t-test (Welch)",0.114064508753959,0.185926133536251,0.999999999999993,0.286046074770879

CAS,"LPD/RL_LPD/SM",0.184513972333333,1.04041666666667,0.855902694333333,"t-test (Welch)",0.11831663043216,0.185926133536251,0.999999999999993,0.586039229564376

PERK4,"LPD/RL_LPD/SM",0.523402861,0.140208341666667,-0.383194519333333,"t-test (Welch)",0.175961437178581,0.241946976120549,0.128686973427825,0.765814006148416

NFY-C9,"LPD/RL_LPD/SM",0.471736194333333,-0.203124991666667,-0.674861186,"t-test (Welch)",0.0698694443529347,0.177882316414115,0.639930056839016,0.797927410127187

NFXL2,"LPD/RL_LPD/SM",0.476736194666667,0.203541675,-0.273194519666667,"t-test (Welch)",0.267841935075147,0.327362365091846,0.369273223170959,0.757105305000766

PYL8,"LPD/RL_LPD/SM",0.726459166666667,0.3375,-0.388959166666667,"t-test (Welch)",0.0676072599370309,0.177882316414115,0.999999999999993,0.482164962724776

CPK33,"LPD/RL_LPD/SM",-0.132152694333333,-0.0611805473333334,0.070972147,"t-test (Welch)",0.882293586811505,0.882293586811505,0.248708178038531,0.796468852828161

ORTHRUS2,"LPD/RL_LPD/SM",0.0845139723333333,1.14375,1.05923602766667,"Wilcoxon",0.0808555983700523,0.177882316414115,0.999999999999993,0.0196896200936435

NAC029,"LPD/RL_LPD/SM",0.389513972333333,1.28041666666667,0.890902694333333,"t-test (Welch)",0.024661295057451,0.177882316414115,0.999999999999993,0.287726227486462

**Supplementary Table S6.** Statistical summary of RT–qPCR gene expression analyses. For each candidate gene, pairwise comparisons were performed between (i) control and drought treatments within each graft combination (LP^C^/RL_LP^D^/RL; LP^C^/SM_LP^D^/SM) and (ii) the two graft combinations under the same treatment (LP^C^/RL_LP^C^/SM; LP^D^/RL_LP^D^/SM). Expression values were analyzed as log₂ fold changes (log₂FC). The Shapiro-Wilk test was used to assess normality of replicate distributions; if both groups were normally distributed (p > 0.05), Welch’s t-test was applied, otherwise a Wilcoxon rank-sum test was used. Reported values include mean log₂FC per group, mean differences, p-values, adjusted p-values (Benjamini–Hochberg correction), and Shapiro–Wilk p-values for each group. No statistically significant differences were observed after multiple testing correction (FDR < 0.05).

| Promoters |  |  |  |  |  |  |  |  |  |  |  |  |  |  |  |  |
| --- | --- | --- | --- | --- | --- | --- | --- | --- | --- | --- | --- | --- | --- | --- | --- | --- |
| **Comparison** | **Methylation contexto** | **Chromosome** | **DMR_start** | **DMR_end** | **DMR_q-value** | **DMR_mean_methylation _difference** | **Number of Cs in the DMR** | **DMR_pvalue (MWU)** | **DMR_pvalue(2D KS)** | **Mean_meth_LC** | **Mean_meth_LD** | **Promoter_start** | **Promoter_end** | **Promoter_strand** | **Gene_name** | **Gene_ID** |
| LC_LD | CHH | NC_068556.1 | 6677014 | 6677156 | 0.043188 | 0.172324 | 32 | 2.14E-08 | 0.00043045 | 0.46509 | 0.29277 | 6676778 | 6678778 | + | PERK4 | LOC102610683 |
| LC_LD | CHH | NC_068558.1 | 34509522 | 34509547 | 0.0014698 | 0.110012 | 8 | 4.87E-07 | 1.51E-06 | 0.11843 | 0.008418 | 34508986 | 34510986 | - | CAS | LOC102623612 |
| LC_LD | CHH | NC_068559.1 | 1162518 | 1162528 | 0.028157 | 0.199034 | 5 | 0.000457 | 0.00019185 | 0.39443 | 0.19539 | 1161066 | 1163066 | + | NFXL2 | LOC102629496 |
|  |  |  |  |  |  |  |  |  |  |  |  |  |  |  |  |  |
| LC_LD | CHH | NC_068558.1 | 34502099 | 34502131 | 0.043188 | 0.267681 | 5 | 0.000174 | 0.00043398 | 0.36118 | 0.093497 | 34497284 | 34508986 | - | CAS | LOC102623612 |
| LC_LD | CHH | NC_068564.1 | 27287301 | 27287335 | 0.025068 | 0.235694 | 8 | 1.64E-05 | 0.00015817 | 0.31847 | 0.082773 | 27250491 | 27310982 | + | CPK33 | LOC102630343 |
| LC_LD | CHH | NC_068564.1 | 27297435 | 27297678 | 5.63E-05 | 0.144433 | 49 | 7.24E-14 | 1.06E-08 | 0.5294 | 0.38497 | 27250491 | 27310982 | + | CPK33 | LOC102630343 |
